# The SGNH hydrolase family: a template for carbohydrate diversity

**DOI:** 10.1093/glycob/cwac045

**Published:** 2022-07-23

**Authors:** Alexander C Anderson, Stefen Stangherlin, Kyle N Pimentel, Joel T Weadge, Anthony J Clarke

**Affiliations:** Department of Molecular and Cellular Biology, University of Guelph, Guelph N1G2W1, Canada; Department of Chemistry & Biochemistry, Wilfrid Laurier University, Waterloo N2L3C5, Canada; Department of Molecular and Cellular Biology, University of Guelph, Guelph N1G2W1, Canada; Department of Biology, Wilfrid Laurier University, Waterloo N2L3C5, Canada; Department of Molecular and Cellular Biology, University of Guelph, Guelph N1G2W1, Canada; Department of Chemistry & Biochemistry, Wilfrid Laurier University, Waterloo N2L3C5, Canada

**Keywords:** carbohydrate acetyltransferase, carbohydrate deacetylase, carbohydrate esterase, CAZyme structure–function, CE subfamilies

## Abstract

The substitution and de-substitution of carbohydrate materials are important steps in the biosynthesis and/or breakdown of a wide variety of biologically important polymers. The SGNH hydrolase superfamily is a group of related and well-studied proteins with a highly conserved catalytic fold and mechanism composed of 16 member families. SGNH hydrolases can be found in vertebrates, plants, fungi, bacteria, and archaea, and play a variety of important biological roles related to biomass conversion, pathogenesis, and cell signaling. The SGNH hydrolase superfamily is chiefly composed of a diverse range of carbohydrate-modifying enzymes, including but not limited to the carbohydrate esterase families 2, 3, 6, 12 and 17 under the carbohydrate-active enzyme classification system and database (CAZy.org). In this review, we summarize the structural and functional features that delineate these subfamilies of SGNH hydrolases, and which generate the wide variety of substrate preferences and enzymatic activities observed of these proteins to date.

## 1. Introduction

Carbohydrate-active enzymes (CAZymes) are responsible for glycan biosynthesis, modification, recognition and turnover that together make up the glycocode (i.e. the genomic resource pool) for a particular organism. CAZymes are divided into groups with differing enzymatic activities. Glycosyltransferases (GTs) are enzymes that catalyze the formation of glycosidic bonds between monosaccharide units to form oligo- or polysaccharides (Breton et al. 2006). Glycoside hydrolases (GHs), by contrast, are responsible for the hydrolysis of these glycosidic bonds during glycan degradation or turnover (Davies and Henrissat 1995). Each of these have been studied extensively and have a clear relationship to glycan diversity, and have been reviewed expertly by others (Davies and Henrissat 1995; Breton et al. 2006; Lairson et al. 2008). In both cases, CAZymes that possess these activities can be further classified into unique families under the carbohydrate-active enzyme (CAZy) and Pfam classification systems and databases (www.cazy.org; pfam.xfam.org) (Cantarel et al. 2009; Lombard et al. 2014; El-Gebali et al. 2019). These protein families are delineated by significant sequence similarity and, because sequence similarity strongly suggests folding similarity (Chothial and Lesk 1986), the members of a single family adopt similar three-dimensional characteristics (Henrissat 1991). The Pfam and CAZy classification systems are therefore distinct from the EC classification system that classifies proteins instead by both their particular enzymatic activities and substrate specificities. As a consequence, protein families often describe a collection of evolutionarily-related sequences that include orthologs, paralogs, and those that have arisen via convergent evolution (Henrissat 1991; Koonin 2005).

Beyond simply the formation or breakage of glycosidic bonds, the modification of glycans at specific residues can further modulate the biological role of glycans, similarly to proteins and nucleic acids. For example, sulfation, acylation, methylation, epimerization, phosphorylation, or a combination of these can occur at various positions in a glycan (Muthana et al. 2012). The important and diverse roles of these modifications are well established in a wide array of biological processes spanning all domains of life (Yu and Chen 2007; Muthana et al. 2012). These carbohydrate modifications and/or their removal have demonstrated roles in many biological processes of research importance, including (i) the biosynthesis of cell wall materials in plants, fungi and bacteria; (ii) the metabolism of plant cell wall materials by the ruminant microbiome and (iii) in the establishment or persistence of infectious disease (Yu and Chen 2007; Muthana et al. 2012; Whitfield et al. 2015). For these reasons, there is significant research interest in identifying discrete carbohydrate modifications and the CAZymes that are responsible for them.

### 1.1 The SGNH hydrolase superfamily

**Fig. 1:**
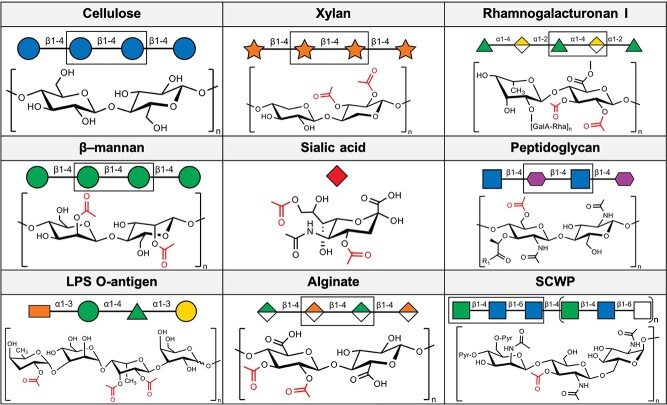
Known substrates of the carbohydrate-active SGNH hydrolase superfamily members. Important cell-wall, cell-surface and extracellular matrix polysaccharides derived from plants, animals and bacteria are both substituted and de-substituted by enzymes of this superfamily found in all domains of life. Symbols are drawn according to the Symbol Nomenclature for Glycans (SNFG; Varki et al. 2015; Neelamegham et al. 2019) using GlycoGlyph (Mehta and Cummings 2020). R_1_ = peptidyl substituent.

The SGNH hydrolase superfamily contains 16 member families (Table 1) and includes >89,000 total members at present. This large and diverse superfamily contains a wide variety of enzymatic activities distributed across all domains of life (Fig. 1). Many members of the SGNH hydrolase superfamily are also classified under the CAZy classification system. Presently, there are 18 CE families contained in the CAZy classification system, numbered 1–9 and 11–19 owing to the withdrawal of the former CE10 that was found to act predominantly on non-carbohydrate substrates. Of these, 15 families contain at least one member that has been structurally characterized with a known or implied substrate preference and catalytic mechanism (Lombard et al. 2014). However, because only carbohydrate esterases (CEs) are defined and classified under this system, only select members of the SGNH hydrolase superfamily are classified under both systems. Select SGNH hydrolase superfamily members can be found distributed across CE families 2, 3, 6, 12 and 17. For the purpose of this review, we focus on carbohydrate-active SGNH hydrolase families as defined under the Pfam classification system.

**Table 1:** Summary of SGNH hydrolase families studied in the literature

Family	Membership	Known activity(ies)	Known structure(s)
AlgX	1090	Alginate *O*-acetyltransferase	2
DHHW	766	SCWP O-acetyltransferase	2
DltD	403	Lipoteichoic acid D-alanyl transferase	3
DUF1574	97	None	0
DUF4886	247	None	1
GSCFA	1242	None	0
Hema_esterase	22	Sialic acid acetylesterase	10
Lipase_GDSL_1	24,410	Acetylxylan esterase Rhamnogalacturonan acetylesterase	10
Lipase_GDSL_2	38,435	Acetylxylan esterase Mannan acetylesterase	45
Lipase_GDSL_3	1219	None	1
Lipase_GDSL_like	46	None	2
OSK	65	RNA-binding	1
PatB (DUF459)	238	Peptidoglycan *O*-acetyltransferase	0
PC-Esterase	10,956	Xylan *O*-acetyltransferase	1
SASA	6189	Sialic acid acetylesterase	3
SGNH_AT3	3702	Peptidoglycan *O*-acetyltransferase Lipopolysaccharide *O*-acetyltransferase	3

Prior to the delineation of the SGNH hydrolase superfamily and the widespread availability of high-resolution crystal structures, many esterases and lipases were once thought to belong to a single superfamily. This family, the α/β hydrolase family (Hemilä et al. 1994; Jaeger et al. 1994), was thought to also share a common fold and overall architecture. In 1995, Upton and Buckley proposed that a subset of these α/β hydrolases actually represented a new and distinct family of lipases and esterases (Upton and Buckley 1995). Their delineation was based on the presence of five blocks (termed simply Blocks I-V) of highly conserved residues within their sequences. The most notable of these is a catalytic GDSL motif that aligned to, but differed from, the GXSXG consensus motif found in α/β hydrolases, and so these enzymes became initially referred to as the GDSL family of lipases and esterases. Although a seemingly trivial difference, the GXSXG motif of known α/β hydrolase structures facilitates its folding into a “nucleophilic elbow,” characterized by a sharp turn between a β-strand and an α-helix. The local folding of this particular consensus sequence forces the backbone of the loop presenting the catalytically important Ser nucleophile to adopt a strained conformation. As such, the presence of a GDSL motif would plausibly prevent a similar local folding and would create a different active site.

Indeed, as the first structures of these newly delineated GDSL enzymes were resolved, it became known this motif folded into a type I β turn that in fact presented the catalytic Ser nucleophile as part of an unstrained loop (Wei et al. 1995; Mølgaard et al. 2000). These early crystal structures also contributed knowledge of other unique features that delineated this fold and the functional role of each of the conserved blocks. While the α/β hydrolase fold typically consists of a central eight-stranded parallel β-sheet flanked by α-helices, the GDSL family possessed a smaller central five-stranded parallel β-sheet with a flavoprotein-like fold (Fig.3). In both families, although the location of these residues differs, a canonical Ser-His-Asp catalytic triad is found. The α/β hydrolases present a catalytic Ser at the C-terminal end of the fifth β-strand, an Asp at the C-terminal end of the seventh β-strand, and a His in a loop region linking the eighth β-strand and the C-terminal α-helix. In contrast, the GDSL family presents the equivalent Ser residue on the C-terminal of the first β-strand in the Block I consensus sequence. The important Asp and His are spaced only three residues apart, presented by a loop between two α-helices on the C-terminal side of the central β-sheet in the conserved Block V. The remaining Block II and Block III residues present strictly conserved hydrogen-bond donors to the oxyanion hole: the amide N atom from a Gly in Block II and the N^δ2^ atom from an Asn in Block III. The remaining Block IV does not contain a catalytically important residue, but instead folds into an α-helix linking β-strands 4 and 5 that is structurally important to the overall fold, rationalizing its conservation. With the identification of the essential catalytic residues, the name “SGNH hydrolase family” was proposed in reference to the strictly conserved Ser, Gly, Asn and His residues found in Blocks I, II, III and V, respectively. This name also accounted for the observation that this new family contained not only lipases as was originally proposed, but that the discovered activities also included the hydrolysis of a variety of ester-linked substituents on various biomolecules as the family’s membership was expanded.

**Fig. 2:**
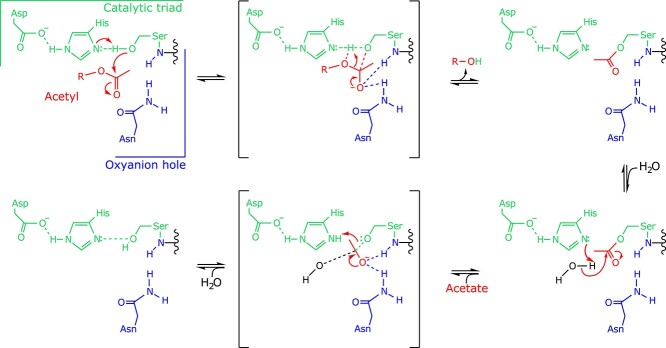
The general SGNH hydrolase mechanism of action. The nucleophilic attack of the ester carbonyl (red) is accomplished by the action of a Ser-His-Asp triad (green), and the resulting oxyanion intermediate species is stabilized by a positively charged pocket often referred to as the oxyanion hole (blue), coupled with the release of the alcohol coproduct. A covalent acetyl-enzyme intermediate is formed during the catalytic cycle, which is then attacked by a water molecule to generate the acetate coproduct and free enzyme.

The generally understood SGNH hydrolase mechanism (Fig. 2) involves a nucleophilic attack of the carbonyl carbon on the ester substrate by this highly nucleophilic Ser residue. The resulting tetrahedral oxyanion intermediate formed by this step of the mechanism is stabilized by a positively charged pocket, termed the oxyanion hole, which is shaped by the backbone amides of the Block I Ser and Block II Gly residues, and the sidechain amide of the Block III Asn residue. The formation of a covalent acyl-enzyme intermediate has been observed and is generally accepted to be a feature of the SGNH hydrolase mechanism, while the resulting oxyanion product is protonated by the enzyme, presumably via the catalytic His, to form the alcohol coproduct. In the subsequent step, a water molecule freely approaches the covalent acyl-enzyme intermediate and is deprotonated by the basic His, initiating a nucleophilic attack of the carbonyl carbon. In a similar fashion, the resulting oxyanion intermediate is stabilized by the oxyanion hole and collapses to form the acyl coproduct and the free enzyme, completing the catalytic cycle.

Although the terms Lipase-GDSL family and SGNH hydrolase family have been used interchangeably (often written as SGNH/GDSL family), it is now generally understood that the term “SGNH hydrolase” is meant to refer to the superfamily or clan of proteins (Pfam: CL0264) that share this particular overall fold, of which the original GDSL family now comprises four of sixteen currently annotated families. At present, five of the SGNH hydrolase families do not have a member with known function: The GSCFA family [PF08885]; DUF1574 family [PF07611]; DUF4886 family [PF16227]; Lipase_GDSL_3 family [PF14606]; and the Lipase_GDSL_like family [PF16255]. At least one crystal structure has been solved in three of these uncharacterized families: the DUF4886, Lipase_GDSL_3 and Lipase_GDSL_like families. Of the eleven families with at least one biochemically characterized member, only two families are not known to contain carbohydrate-active enzymes. These are the OSK family (PF17182); which are RNA binding domains involved in germline development; and the DltD family (PF04914), which are d-alanyl transferases involved in bacterial lipoteichoic acid biosynthesis. The remaining nine families contain, at least in part, CAZymes among their members which catalyze a wide variety of biologically important carbohydrate modifications. Although it is worth mentioning that many carbohydrate-active enzymes belonging to the SGNH superfamily remain CAZy-unclassified, members of these families also belong to the CE families 2, 3, 6, 12 and 17 under the CAZy classification system.

In the 26 years since the SGNH hydrolase family fold was first described, a wealth of available structural and functional data has shown that the general architecture of this fold serves as a template for a wide variety of carbohydrate-modifying enzymes that span the kingdoms of life. In this review, we will focus on recent structural and functional insight into CAZymes that belong to the SGNH hydrolase superfamily. With a particular focus on structure and function, we highlight that the highly conserved SGNH hydrolase fold among this superfamily serves as a structural template for the wide variety of known carbohydrate-modifying enzymes. These modifying enzymes commonly display either *O*-acetylesterase or *O*-acetyltransferase activities on a diverse range of glycans, and their description informs the basis of existing CE families and provides insight into the evolution of this understudied class of CAZymes.

### 1.2 The Lipase_GDSL families

Among the largest of the families are the GDSL-like lipase/acylhydrolases, comprising 51,305 sequences of the 71,599 total presently belonging to the SGNH hydrolase superfamily. These enzymes are annotated into four distinct Pfam families: GDSL-like lipase/acylhydrolase 1 (GDSL-1), GDSL-like lipase/acylhydrolase 2 (GDSL-2), GDSL-like lipase/acylhydrolase 3 (GDSL-3) and GDSL-like lipase/acylhydrolase like (GDSL-like) (Pfam.org); however, a vast majority of these sequences belong to the GDSL-1 (17,037 sequences) and GDSL-2 (32,197 seq.) families. Accordingly, only members from these two families have been biochemically characterized, though structures are available on the protein data bank (PDB) for both the GDSL-3 and GDSL-like families. Members of this group are comprised primarily of sequences from bacteria (27,721 seq.), plants (14,724 seq.) and fungi (6047 seq.), with the remainder belonging to metazoans (1509 seq.), viral genomes (219 seq.), archaea (37 seq.) and others (4 seq.). Interestingly, this taxonomic distribution is asymmetrical across the families, with the GDSL-1 family being composed chiefly of sequences belonging to plants (13,507 seq., 79 percent), while the GDSL-2 family is composed primarily of bacterial sequences (25,556 seq., 79 percent). All of the biochemically characterized and structurally resolved enzymes belonging to these GDSL families belong to bacterial and fungal microorganisms and have a demonstrated or putative role in metabolism of plant cell wall polysaccharides.

#### 1.2.1 Xylan esterases

Hemicelluloses, first identified as alkali extracts of plant cell walls (Clayson et al. 1921), are a vital group of polymers for plants to maintain cell wall integrity and growth (Lucenius et al. 2019). From these polysaccharides, xylan is one of the primary members of this group and is among the most abundant biopolymers on Earth, second only to cellulose (Fig. 1). Xylan is composed of β (1 → 4) xylosyl residues, and may possess a variety of functional groups, including acetyl esters which sterically preclude the activity of GHs on the polymer and make them poor GH substrates (Cantarel et al. 2009). To circumvent this, acetyl xylan esterases (AXEs) deacetylate xylan, ultimately improving the access of related GH enzymes with specific preference for deacetylated xylan substrates (Poutanen et al. 1990).

Many bacterial GDSL sequences are characterized by a conserved domain architecture consisting of an N-terminal carbohydrate-binding module (CBM) domain and a C-terminal SGNH hydrolase domain (Montanier et al. 2009). Although sequences with this domain architecture are split between the GDSL-1 and GDSL-2 families, the CAZy classification groups enzymes with this domain architecture exclusively in CE2. The characteristic CBM domain found in CAZy CE2-classified GDSL members is itself a member of the CBM family 1 (CBM1; Montanier et al. 2009), and is likely not directly involved in catalysis but rather responsible for facilitating carbohydrate binding and shaping substrate preference of the catalytic domain (Till et al. 2013). Typically, the N-terminal CBM domains found in these sequences are composed of a jelly roll fold, containing two β-sheets with four and five antiparallel β-strands. The C-terminal catalytic domains of these enzymes then belong to the GDSL-1 and GDSL-2 families (Mølgaard et al. 2000; Montanier et al. 2009), possessing the typical SGNH hydrolase fold. As is typical of SGNH hydrolase family members, the catalytic architecture of the CE2 C-terminal domain is presented by four conserved loops of Blocks I, II, III and V (Till et al. 2013). While all GDSL/CE2 enzymes that have been structurally resolved possess a typical oxyanion hole composed of the Block II Gly and Block III Asn, many GDSL enzymes that also belong to CAZy family CE2 notably are not thought to possess the typical Ser-His-Asp catalytic triad but instead a Ser-His catalytic dyad (Till et al. 2013). The unusual presence of a Ser-His dyad among CE2 enzymes most likely corresponds to the presence of an additional Trp residue in the Block V loop. This Trp is partially conserved, particularly among members of CAZy family CE2: multiple sequence alignment suggests that 14 of 26 CE2 sequences examined by Montanier et al. (2009) do not align with an Asp residue at the conserved Block V site. However, this feature does not appear to serve as a basis for Pfam classification, as these CAZy CE2 sequences are distributed among both GDSL-1 and GDSL-2.

*Clostridium thermocellum* CE2 (*Ct*CE2; Montanier et al. 2009) is a member of families GDSL-1/CE2 with an experimentally resolved structure (PDB id 2WAO). The structure of *Ct*CE2 validates that the aforementioned equivalent Trp residue in this enzyme displaces the conserved Block V loop and forces the Asp residue, which would normally function in the catalytic triad, into a distorted conformation that prevents it from interacting with the conserved Block V His (Fig. 4). This structural insight demonstrated that indeed this molecular feature disrupts the otherwise conserved Block V fold found in all other structurally resolved GDSL family enzymes to date (Montanier et al. 2009).

**Fig. 3:**
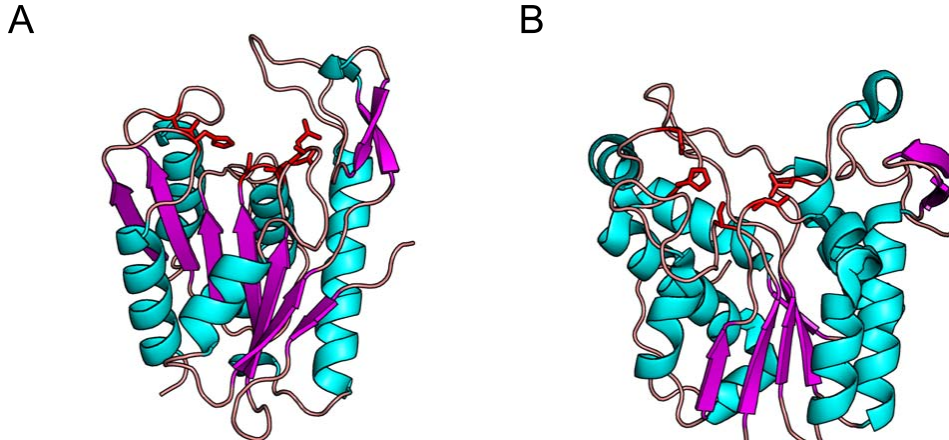
The protoctypical SGNH (A; *Aa*Rha1, 1DEO) and α/β hydrolase (B; *Pseudomonas fluorescens* carboxylesterae, 1AUO) folds. Although both superfamilies adopt an α/β/α sandwich fold of similar size, the key catalytic residues (red) and their placement differ. In the SGNH hydrolase superfamily, the catalytic acid/base pair are contained together on a single loop and the oxyanion hole is usually lined by an Asn and Gly, which donate H-bonds. In the α/β hydrolase superfamily, the acid and base are presented by separate loops, and the oxyanion hole is lined by neighboring backbone amide groups as H-bond donors.

**Fig. 4:**
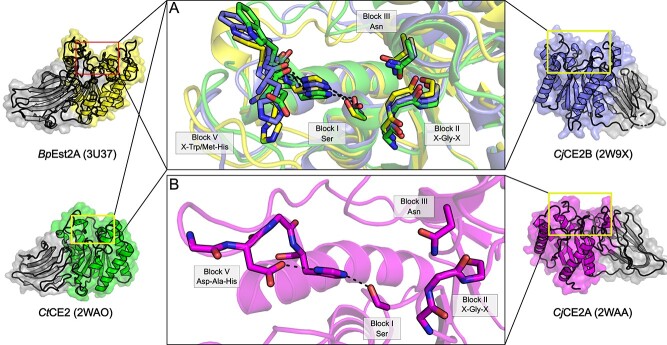
GDSL/CE2 family enzymes. Representative structures of (A) the putative bacterial acetylxylan esterases *Bp*Est2A (3U37; yellow), *Cj*CE2B (2W9X; blue), *Ct*CE2 (2WAO; green) and (B) *Cj*CE2A (2WAA; magenta), which contain appended CBM domains. A catalytic dyad lacking the typically conserved Block V Asp residue is observed in many bacterial enzymes with this domain architecture, although not exclusively, as exemplified by *Cj*CE2A.

The presence of a dyad is also unusual among other more distantly related serine hydrolases, as the catalytic nucleophile/base pair Ser/His are classically accompanied by an Asp or Glu residue, which enhance the nucleophilicity of the catalytic Ser, and thus are essential for the catalytic efficiency in these enzymes (Mølgaard et al. 2000). In place of the family-typical acidic residue at this location, structural studies of CE2 esterases demonstrate that tuning of the nucleophile/base pair Ser/His is instead accomplished though hydrogen bonds with adjacent main chain carbonyl groups (Fig. 4; Montanier et al. 2009, Till et al. 2013).

In addition to their role in shaping the characteristic CE2 catalytic dyad, aromatic residues are also crucial in both substrate recognition and binding among CAZymes as first demonstrated with lysozyme (Phillips 1967). In at least one case, the structurally resolved GDSL-2/CE2 enzyme Est2A from *Butyrivibrio proteoclasticus* (*Bp*Est2A; 3U37; Till et al. 2013) contains an active site found near the bottom of a groove shaped primarily by two Trp residues in line with the active site (Till et al. 2013). This groove spans the surface of the catalytic C-terminal SGNH domain, akin to the substrate binding cleft of many GH enzymes and is positioned ideally for the accommodation of an incoming polysaccharide substrate (Till et al. 2013). At least one of these two residues is conserved, with an equivalent Trp or Tyr present in many CE2 sequences.

Unlike *Ct*CE2 and *Bp*Est2A, the structurally resolved GDSL-2/CE2 enzyme, CE2A from *Cellvibrio japonicus* (2WAA; Montanier et al. 2009), does not contain a catalytic Ser/His dyad, but instead employs the usual Ser-His-Asp triad expected of SGNH hydrolases (Mølgaard et al. 2000; Montanier et al. 2009), making it a notable exception. Additionally, of the twelve enzymes surveyed by Montanier and colleagues which still possess the Ser-His-Asp triad at the conserved locations, ten contain a Trp residue as part of the Block V DXXH motif, presumably displacing the Asp sidechain as seen in *Ct*CE2 (Montanier et al. 2009). The final two GDSL/CE2 enzymes instead contained an Ala between the putative catalytic Asp and His residues (Montanier et al. 2009). Despite these anomalies, the proteins remain classified in CAZy CE2 owing to the presence of the N-terminal CBM1 domain characteristic of the family (Montanier et al. 2009). While not structurally confirmed, presumably the displacement and/or loss of the Block V Asp is a common catalytic feature of CE2 that delineates it from other CE families, although this is not without exception, e.g. with *Cj*CE2A (Montanier et al. 2009). Taken together, these studies suggest a substrate preference guided by the N-terminal CBM domain and/or a substrate binding-groove across the surface of the C-terminal SGNH domain, which are also defining features of the CE2 family (Till et al. 2013).

In other work, the GDSL-1/CE3 enzyme BnaC, from the ruminal fungus *Neocalimastix patriciarum* (*Np*BnaC), displayed hydrolytic activity towards acetylated birchwood xylan, as well as several cell-wall derived materials and many acetylated naphthyl derivatives (Dalrymple et al. 1997). While *Np*BnaC does not display specificity for α- or β-linked substituents, there is a clear preference for shorter chain fatty acids (acetyl > > propionyl > butyl, capryl). A dockerin-like domain appended to the C-terminal of *Np*BnaC along with a family 10 CBM strongly suggests the enzyme functions as part of a larger cellulosome complex involved in the hydrolysis of complex plant cell-wall polymers (Dalrymple et al. 1997). Over the past few decades, the evidence for a cellulosome in anaerobic fungi has been expanding (Wilson and Wood 1992; Matthews et al. 2019), including for that of *Neocallimastix* spp., and is found comprised mainly by CAZymes (Fanutti et al. 1995; Haitjema et al. 2017). However, further biochemical characterization of *Np*BnaC is still needed to understand its specific role in the fungal cellulosome.

Two additional enzymes were first identified from the cellulolytic anaerobic rumen bacterium *Ruminococcus flavefaciens* strain 17 and belong to GDSL-1/CE3, (*Rf*CesA) and GDSL-2/CE3 (*Rf*XynB). Both of these enzymes demonstrated the ability to hydrolyze chemically acetylated xylan and native steam-extracted acetylated xylan *in vitro*, as well as other common cell-wall derived materials and acetylated naphthyl derivatives (Zhang et al. 1994; Aurilia et al. 2000). Both enzymes had the highest preference for the α-linked substrate (α-naphthyl acetate; α-NAc) and shorter chain fatty acids (acetyl > propionyl), but further testing is needed to identify their natural substrates given that the panel of substrates assessed were commercial oligomers. Interestingly, synergy was found between *Rf*CesA and a CE15 glucuronoyl esterase, which is proposed to hydrolyze the ester linkage between lignins and glucuronoxylans (Aurilia et al. 2000), as well as between *Rf*XynB and a family 11 glycoside hydrolase (GH11) endoxylanase, which is responsible for degrading xylan polysaccharides (Zhang et al. 1994). Whereas both *Rf*CesA and *Rf*XynB were shown to be capable of hydrolyzing linkages on a xylan substrate, the enhanced esterase activity observed together with appended GH11 and CE15 domains, respectively, suggests that these esterases may have a similar activity profile regardless of substrate length. This also indicates that the GDSL/CE3 enzymes may be more active in de-acetylating oligosaccharides produced by the GH11 and CE15 domains compared to polysaccharides.

Similarly, the GDSL-2/CE3 member Ces3–1 from *C. thermocellum* (*Ct*Ces3–1), was biochemically characterized in vitro and lacked the ability to hydrolyze common CE-family substrates and cell-wall derived materials: aryl-ferulates, aryl-coumarates, acetylated glucomannans, *N*-acetylglucosamine, Cephalosporin C and acetyl deoxyadenosine (Correia et al. 2008). However, *Ct*Ces3–1 is able to hydrolyze *p*-nitrophenyl acetate (*p*NP-Ac), acetylated birchwood xylan and oligosaccharides derived from acetylated xylans by a GH11 xylanase (Correia et al. 2008), suggesting specific activity of *Ct*Ces3–1 towards acetylated oligomeric and polymeric xylans, which is consistent with a presumptive biological role in degrading the plant cell wall. Additionally, *Ct*Ces3–1 displayed significant activity towards α-NAc, similar to results found with other CE3 enzymes.

Akin to *Ct*Ces3–1, the GDSL-2/CE3 enzyme AE206 from the fungus *Talaromyces cellulolyticus* (*Tc*AE206), displays activity towards *p*NP-Ac and acetylated oligosaccharides, as well as *p*-nitrophenyl butyrate (*p*NP-Bu), but not acetylated xylans (Watanabe et al. 2015). A similar trend was found to other GDSL-2/CE3s with a preference of *Tc*AE206 activity towards shorter chain fatty acids (acetyl > > butyl > octanoyl). A direct comparison of the catalytic efficiency (*k*_cat_/*K*_m_) can be noted for acetylesterase activity with *p*NP-Ac, where values of 152.7 and 44.7 mM^−1^ s^−1^ for *Ct*Ces3–1 and *Tc*AE206, respectively, have been reported (Correia et al. 2008; Watanabe et al. 2015).

While both enzymes were characterized as truncations lacking their respective accessory domains or binding partners (Type I dockerin in *Ct*Ces3–1; Correia et al. 2008, CBM1 in *Tc*AE206; Watanabe et al. 2015), differences in substrate preference and catalytic efficiency could be attributed to subtle structural differences near their respective active sites, as well as different structural features that can also lead to preferences of conditions, such as that of pH and temperature. As reported for *Tc*AE206, the optimal pH and temperature for the hydrolysis of *p*NP-Ac is 6.0 and 65 °C, respectively, with some activity between pH 5.0–8.0 and no activity measured above 75 °C (Watanabe et al. 2015). Similarly, optimal thermostability and biophysical properties of *Ct*Ces3–1 were shown to be at a pH of 7.0 and up to a temperature of 70 °C, with loss of activity above 70 °C (Correia et al. 2008).

**Fig. 5:**
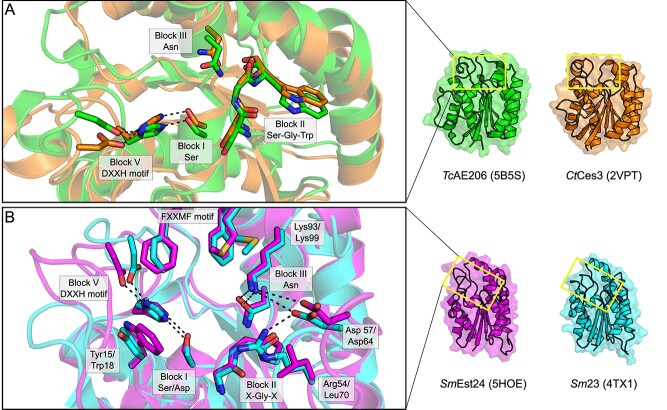
Single-domain GDSL/CE3 family enzymes display heterogeneity of the active site pocket. (A) The active site of two characterized AXEs from *Tallaromyces cellulolyticus*, TcAE206 (5B5S; lime) and from *C. thermocellum, Ct*Ces3 (2VPT; orange) possess an active site fold typical of canonical SGNH hydrolase family members. (B) The active site of two presumptive AXEs from *Sinorhizobium melloti, Sm*Est24 (5HOE; magenta) and *Sm*23 (4TX1; cyan) feature an extended loop that presents an amphipathic helix not found in other CE3 members. TcAE206/CtCes3 RMSD 1.5 Å; SmEst24/Sm23 RMSD 0.7 Å.

Of the above five characterized GDSL/CE3 enzymes, only the two GDSL-2/CE3 enzymes *Tc*AE206 (5B5S; Watanabe et al. 2015) and *Ct*Ces3–1 (2VPT; Correia et al. 2008) have been structurally resolved. Both *Tc*AE206 and *Ct*Ces3–1 adopt an (α/ꞵ/α)-sandwich fold typical of the SGNH hydrolase family (Mølgaard et al. 2000) whereby five central parallel ꞵ-strands are flanked by several α-helices and align well with a root mean square standard deviation (RMSD) of 1.5 Å across 192 equivalent Cα atoms (Fig. 5A). The ability of *Tc*AE206 to accommodate longer-chain linear saccharides, as compared to *Ct*Ces3–1, is rationalized by an overall larger volume of the active site pocket in *Tc*AE206, almost 2-fold larger than that of *Ct*Ces3–1. Structural differences in the active site manifest from a loop region in proximity to the active site pocket in *Ct*Ces3–1 (Glu47-Arg58), which contains an additional six amino acids compared to *Tc*AE206 (Glu13-Arg18). The loop of *Ct*Ces3–1 also presents a Met residue (Met49) that is thought to constrain access to the catalytic Ser (Correia et al. 2008; Fig. 5A). However, there is no direct evidence of the potential influence that Met49 has on substrate binding or preference as a consequence of the lack of natural substrates bound in the structural characterization of *Ct*Ces3–1. Further, *Tc*AE206 is described to contain a flatter, and more hydrophobic active site entrance relative to the charged active site of *Ct*Ces3–1. Both enzymes contain negatively charged binding sites; however, *Ct*Ces3–1 contains a deeper negatively charged pocket. These subtle differences proximal to the active site of *Tc*AE206 and *Ct*Ces3–1 likely generate the differing substrate preferences between these enzymes and rationalize the lack of observed activity of *Tc*AE206 towards polysaccharide substrates otherwise recognized by *Ct*Ces3–1 (Correia et al. 2008; Uechi et al. 2016). Notably, a second esterase domain of *Ct*Ces3 is encoded directly after *Ct*Ces3–1 (*Ct*Ces3–2, 97 percent identity, uncharacterized; Correia et al. 2008) on the same polypeptide. If both domains hydrolyze the same xylan chain, this could create an avidity effect, potentially increasing the preference of *Ct*Ces3 esterases for xylan polysaccharides rather than xylooligosaccharides.

Interestingly, both *Tc*AE206 and *Ct*Ces3–1 contain a DFVGX_n_DXD loop motif (residues 35–56 and 73–89 respectively) that coordinates a calcium ion located above the N-terminal end of the central β-strand (β2). This loop is generally conserved across all currently characterized GDSL-2/CE3s. As seen in *Tc*AE206 and *Ct*Ces3–1, the calcium ion is coordinated through electrostatic interactions from the sidechain oxygens of three Asp residues, along with the main chain carbonyl oxygen of a Phe residue and two water molecules. Treatment of *Ct*Ces3–1 with EDTA does not impair function (Correia et al. 2008), so it is presumed that the metal ion plays a role in structural stability rather than catalysis. A coordinated zinc ion was also observed next to a calcium ion in a *Tc*AE206_S10A variant (5B5L; Uechi et al. 2016); however, this was attributed to the use of ZnSO_4_ in the crystallization conditions. The experimental structure of *Tc*AE206 contains a disulfide bond between the cysteine pair Cys16-Cys47 near the N-terminus that is not present in any other characterized GDSL or CE3 enzymes (Watanabe et al. 2015). Although the authors present some evidence that the disulfide may be catalytically important and conserved in some fungal CE3 homologs, (Watanabe et al. 2015) it should be noted that the protein was expressed heterologously and exposed to oxidizing conditions for prolonged periods to facilitate crystallization. Further, the enzymatic rates of *Tc*AE206 and amino acid variants thereof used as evidence for the disulfide bond differ greatly from others reported in the literature. The location of this disulfide bond in *Tc*AE206 plausibly functions to fix the helix which presents the catalytic Ser nucleophile, a feature of other α/β families including the alkaline phosphatase superfamily (Milla 2006). Contextually, it is difficult to understand its relevance given the lack of characterized GDSL-2/CE3 enzymes containing a disulfide bond and the dearth of structural data available for GDSL/CEs in general relative to the size of these families.

Three putative GDSL-2/CE3 enzymes from *Sinorhizobium meliloti* have also been biochemically characterized (*Sm*Est24; Oh et al. 2016, *Sm*23; Kim et al. 2015 and *Sm*AcE1; Oh et al. 2018); however, only artificial substrates were used in this research, and so their biological activity as AXEs remains speculative. Both *Sm*Est24 and *Sm*23 displayed significant acetylesterase activity towards naphthyl derivatives, particularly with preference for α-linkages and shorter chain fatty acids (acetyl > butyl), which agrees with trends of the true esterases. *Sm*AcE1 was not analyzed with naphthyl derivatives, but a qualitative assay did demonstrate activity towards glucose pentaacetate and cellulose acetate, which was also noted for *Sm*Est24 and *Sm*23 (Kim et al. 2015; Oh et al. 2016; Oh et al. 2018). *Sm*Est24 (5HOE; Oh et al. 2016) and *Sm23* (4TX1; Kim et al. 2015) have also been structurally resolved, each containing the (α/ꞵ/α)-sandwich fold with a Ser-His-Asp catalytic triad typical of the SGNH hydrolase family (Fig. 5B). Notably, three loop protrusions that surround the active site pocket are present in both enzymes that are not seen in other characterized GDSL/CE3s (Kim et al. 2015; Oh et al. 2016). In the first loop from *Sm*23, Tyr15 is in close proximity to the Block I Ser and Block V His residues, whereas in *Sm*Est24, an equivalent Trp18 is at this position (Fig. 5B). Interestingly, both of these residues in their respective enzymes are positioned similarly to Met49 in *Ct*Ces3–1. Likewise, a second loop containing charged residues Asp57/64, Arg64/69 and Lys93/99, in *Sm*23/*Sm*Est24, respectively surrounding the Block III Asn, are presumed to create an H-bonding network that helps stabilize the oxyanion hole (Fig. 5B). Also, the equivalent Arg69 residue of *Sm*Est24 is displaced by Leu70, which disrupts the extended H-bonding network as seen in *Sm*23. Additionally, hydrophobic residues including Met148/155 and two Phe residues (Phe145,149/Phe152,156), for *Sm*24/*Sm*Est24 respectively, create an FXXMF motif located on an α-helix above the active site and is presumed to help position incoming substrate (Fig. 5B). Together with Tyr15/Trp18, the hydrophobic residues enclose the active site pocket, which may shape a substrate preference for smaller oligosaccharides. Unfortunately, the substrate preferences have not yet been fully explored in the characterization of these putative GDSL-2/CE3 enzymes and so the functional consequences of these loop protrusions remain speculative.

All of the GDSL/CE3 enzymes discussed above (characterized and putative) contain a Block I GXSXT motif that includes the catalytic Ser; however, it remains unclear what the function of the conserved Thr residue has in this position. Additionally, *Sm*Est24 differs from the canonical Block II GXSG motif, instead having an Ala residue in place of the consensus Gly (bolded), albeit the backbone amide is positioned accordingly for proper function.

#### 1.2.2 Rhamnogalacturonan esterases

In addition to xylans, another vital group of complex plant cell wall hemicellulose components are rhamnogalacturonans, which are one of the primary components of pectin (Mohnen 2008). Physiologically, rhamnogalacturonans can be found in the cell walls of gymnosperms and angiosperms (Edashige and Ishii 1997). The most abundant constituent form, rhamnogalacturonan I (Fig. 1), consists of repeating units of (→2) rhamnose α (1 → 4) galacturonic acid (1 →) (Thibault et al. 1993). Like many polysaccharides found in biological systems, rhamnogalacturonan is heavily decorated with chemical modifications which make the carbohydrate recalcitrant to degradation, such as methyl carboxylates at C6 and acetyl esters at either the C2 or C3 hydroxyl position of galacturonic acid residues (Ishii 1997). These modifications sterically hinder the hydrolytic activity of rhamnogalacturonan hydrolases on the α (1 → 4) bonds between rhamnose and galacturonic acid (Schols et al. 1990; 1995). In order to circumvent this resistance, acetyl groups are removed from galacturonic acid residues through the action of rhamnogalacturonan acetylesterases (RGAEs, EC 3.1.1.86; Schols et al. 1990). This activity is unique and exclusive to members of the CAZy classified CE12 family, at least among characterized and classified CEs to date, as reviewed by Nakamura et al. (2017).

**Fig. 6:**
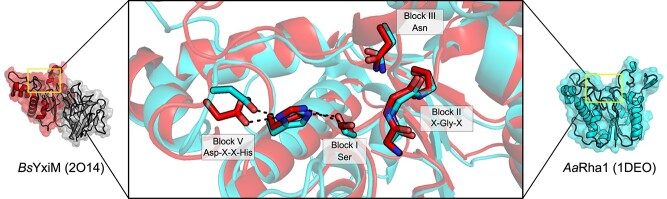
GDSL/CE12 members are the prototypical SGNH hydrolases. The structure of the *A. acelatus* RGAE Rha1 (1PP4; cyan) and the *B. subtilis* YxiM (2O14; red). A catalytic Ser is presented by a type I β turn, and the catalytic acid/base pair are encoded together in the Block V motif, delineating SGNH hydrolases from the α/β hydrolases. The figures were rendered in PyMOL, with an RMSD of 1.4 Å between structures.

The first structure of a GDSL-1/CE12 enzyme to be reported was for that produced by *Aspergillus aculeatus* Rha1 (*Aa*Rha1; 1DEO/1PP4; Mølgaard et al. 2000). *Aa*Rha1 possesses the hallmark SGNH hydrolase domain, with the corresponding conserved amino acids characteristic of the family. In addition, the family also contains a similar α/β hydrolase-like fold in which five parallel β-strands are surrounded by a series of α-helices (Fig. 6). However, in contrast to the nine α-helices reported in other CE family structures, only eight helices contribute to the fold of GDSL/CE12 enzymes (Mølgaard et al. 2000). The active site of *Aa*Rha1 was originally identified from its structure owing to the presence of the typical Ser-Asp-His catalytic hydrogen bonding geometry found in the related α/β hydrolase family. The presence of a bound sulphate ion in the active site pocket of one structure (1DEO) mimics the tetrahedral transition state of an acetyl group and provided important insight on the subtleties that shape the features of the SGNH hydrolase family. The backbone amides of the Block I Ser and Block II Gly residues, together with the sidechain amide of the Block III Asn residue, make polar contacts with the sulphate oxygen, forming the oxyanion hole (Mølgaard et al. 2000). In addition, another sulphate oxygen atom interacts with the conserved Block V His imidazole ring (Mølgaard et al. 2000). Further examination of *Aa*Rha1 suggests that the active site is situated within a large cleft which likely serves in the accommodation of rhamnogalacturonan substrate. In support of this theory, *Aa*Rha1 possesses a series of Arg residues lining this cleft, which may aid in the accommodation of negatively charged carboxylate groups, which are characteristic of rhamnogalacturonan, likely making this cleft a defining feature of CE12 (Mølgaard et al. 2000).

The structure of a second putative RGAE, YxiM from *Bacillus subtilis* (*Bs*YxiM; 2O14) is available, although as a structural genomics target, which lacks biochemical characterization or an associated study in the literature. The structure of *Bs*YxiM demonstrates overall similarity to *Aa*Rha1, with superimposition resulting in a RMSD of 1.5 Å across 134 equivalent Cα atoms with active site residues occupying equivalent positions within each enzyme (Fig. 6). *Bs*YxiM also contains within its structure an appended CBM, related to known galactose-binding domains, which adopts a jelly-roll fold. This feature is apparently shared with other GDSL/CE12 family members, including a putative RGAE from *Bacillus halodurans* (*Bh*RGAE; Navarro-Fernández et al. 2008) and YesT from *B. subtilis* (*Bs*Yes; Martínez-Martínez et al. 2008)*,* though little more has been demonstrated of GDSL/CE12 RGAE enzymes in the literature since.

Most recently, the crystal structure of a GDSL-2/CE17 enzyme from *Roseburia intestinalis* (RiCE17; 6HFZ, 6HH9; Michalak et al. 2020), a prevalent gut commensal bacterium, revealed that it comprises a CBM35 domain appended to an SGNH hydrolase domain. Characterization of the enzyme revealed that it displays 2-*O*-acetylesterase activity on plant cell wall mannans (Fig. 1). *Ri*CE17 was shown to act synergistically with a second GDSL-2/CE2 enzyme, *Ri*CE2, which deacetylates mannans at the 2-, 3-, or 6 positions, requiring deacetylation at the 2-*O*-acetyl position by *Ri*CE17 first for doubly acetylated mannans. This enzyme, although structurally related to other GDSL-2/CE2 enzymes, contains the SGNH domain at its N-terminal and the CBM domain at its C-terminal, where other GDSL-2 and CE-2 enzymes with accessory CBM domains contain these inserted within the SGNH domain, as in Ape1 from *Neisseria meningitidis* (*Nm*Ape1; Williams et al. 2014) or at its N-terminal, as in *Cj*CE2 (Montanier et al. 2009).

#### 1.2.3 Sialic acid-specific acetylesterase family

At the time of writing, the sialic acid-specific acetylesterase (SASA) family consists of 4054 known or putative sequences; a majority belonging to bacteria (3076 seq.), with the remainder belonging to plants (519 seq.), fungi (81 seq.), viruses (54 seq.), animals (191 seq.) and other eukaryota (133 seq.). Many SASA family proteins are also classified under the CAZy database as members of carbohydrate esterase family 6 (CE6).

Although few SASA enzymes have been biochemically characterized, SASA domains are generally understood to function as sialic acid 9-*O*-acetylesterases. Sialic acids are a class of saccharides derived from 5-*N*-acetylneuraminic acid (Neu5Ac), an unusual 9-carbon sugar (Fig. 1; Angata and Varki 2002). In animals, the de-*O*-acetylation of surface sialic acids functions in B cell tolerance and development (Cariappa et al. 2009). Deletion of these esterases induces defects in B cell signaling and peripheral B cell development (Cariappa et al. 2009). Accordingly, the deletion or accumulation of loss-of-function mutations of SASA enzymes in mice results in autoimmunity mediated by dysregulation of B cell receptor signaling.

Studies of the SASA family member NanS in *E. coli* O157:H7 (*Ec*NanS) suggest that bacterial SASA proteins also appear to function as ﻿9-*O*-acetyl Neu5Ac deacetylases, possibly for the purpose of sialic acid catabolism (Rangarajan et al. 2011). While a majority of SASA proteins do not contain appended domains, a small fraction of them do contain predicted glycosyl hydrolase domains, present almost exclusively in bacterial SASA sequences. This observation supports the role of sialic acid degrading SASA enzymes in at least a subset of bacterial species.

In plants, however, the specific roles of these proteins remain to be seen as plant tissues are not known to contain or display sialic acids or other sialosides. In the absence of sialic acids, acetylxylan has been proposed as the natural substrate for some of these SASA proteins in plants and bacteria. In support of this theory, a pair of adjacent SASA enzymes in the rumen-colonizing bacterium *Fibrobacter succinogenes*, Axe6A and Axe6B (*Fs*Axe6A and *Fs*Axe6B), demonstrate synergistic activity with the xylanase Xyn10E (Kam et al. 2005). Though their activity was equal in the presence or absence of the xylanase, only acetylated xylan polysaccharides treated with *Fs*Axe6A or *Fs*Axe6B served as a substrate for the glycosyl hydrolase Xyn10E (Kam et al. 2005). A similar observation was made of Axe2 from the anaerobic rumen fungus *Orpinomyces sp.* strain PC-2 (*Op*Axe2), which showed synergistic degradation of acetylxylan with the xylanase XynA from the same organism (Blum et al. 1999). In both cases, full degradation of acetylxylan was dependent upon the putative AXE activity, strongly suggesting acetylxylan as the natural substrate for these enzymes (Blum et al. 1999; Kam et al. 2005). Interestingly, *Fs*Axe6A and *Fs*Axe6B were capable of binding insoluble cellulose and beechwood xylan and retaining their activities; however, this feature may be attributed to the presence of a family 6 carbohydrate-binding module (CBM6) that is part of these enzymes (Kam et al. 2005).

**Fig. 7:**
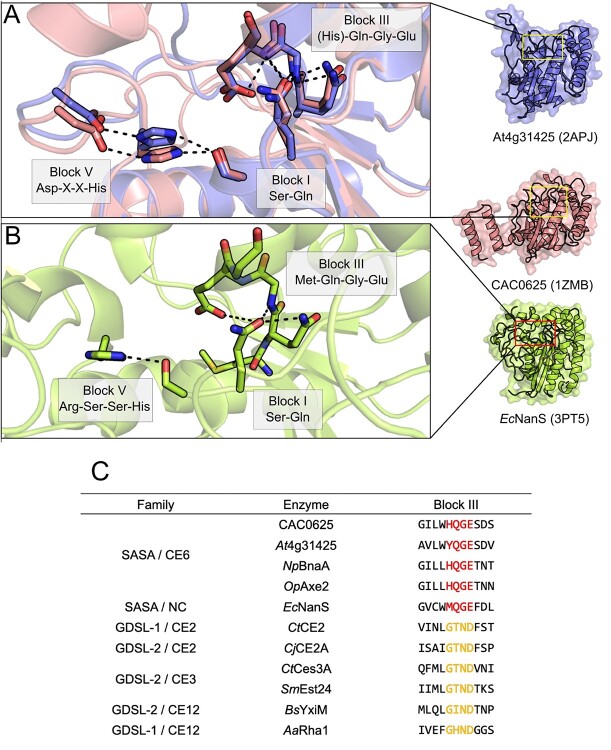
The SASA family enzymes adopt an inverted oxyanion hole structure. (A) The overall structures of *At*4g31425 (2APJ; blue), CAC0625 (1ZMB; salmon) and (B) *Ec*NanS (3PT5; lime) possess a typical SGNH hydrolase fold but an unusual oxyanion hole, where *Ec*NanS lacks the conserved Block V Asp. The conserved (H)QGE motif found in Block III of SASA/CE6 enzymes create a hydrogen bond network that orients a Gln residue locked on Block I inwards and facing the active site. (C) A multiple sequence alignment of representative family members examined here exemplifies the unique Block II (H)QGE motif among SASA/CE6 family enzymes.

To date, three structures of SASA family enzymes have been deposited to the PDB: the structure of the *Arabidopsis thaliana* putative AXE *At*4g34215 (2APJ; Bitto et al. 2005), the bacterial sialic acid esterase *Ec*NanS (3PT5; Rangarajan et al. 2011) and the putative AXE CAC0529 from *Clostridium acetobutyliticum* (1ZMB; Forouhar et al 2005). Interestingly, only *Ec*NanS was functionally characterized and demonstrated maximal activity on the sialic acid 9-*O-*acetyl Neu5Ac (Rangarajan et al. 2011), while the activities of the other putative AXEs remain to be demonstrated. These enzymes adopt an α/β fold typical of the SGNH hydrolase family composed of a central 7-stranded β-sheet bundled between a series of α-helices packed against both sides (Fig. 6A; Bitto et al. 2005). Despite these sequences spanning separate domains of life, they share a near-identical fold, with an average pairwise RMSD of 1.6 Å across at least 153 equivalent Cα atoms when comparing their structures. Only small superficial structural differences are apparent between these structures. The structure of CAC0529 contains an extended C-terminal region as compared to *At*4g34215, which folds into a series of three α-helices not packed against the central SGNH domain (Fig. 7A). While this C-terminal region may be logically associated with *C. acetobutyliticum* cellulosome complex integration, this C-terminal region does not fold into a known dockerin structure, and an understanding of its role would require biochemical characterization. By contrast, *At*4g34215 contains an extended loop between Gly38 and Asp51, as compared to CAC0529, which instead contains a short, 2-stranded antiparallel β-sheet fold in proximity to the active site, an unusual feature among SGNH hydrolase family members (Bitto et al. 2005). *Ec*NanS contains a series of extended loop structures which fold back towards the C-terminal face of the enzyme. In all three enzymes, the central conserved SGNH hydrolase domain is otherwise near-identical.

A key distinguishing characteristic of SASA family enzymes is their unusual so-called Block III sequence that differs greatly from other SGNH hydrolase family sequences (Fig. 7B). While classically this motif presents an asparagine residue to the oxyanion hole, the Block III motif in SASA family members does not contain an equivalent conserved asparagine residue (López-Cortés et al. 2007). Instead, the available SASA structures present a glutamine residue, adjacent to the catalytic serine, which is positioned in the oxyanion hole by a hydrogen bond network with the conserved SASA Block III HQGE motif (Fig. 7; Bitto et al. 2005). This results in an inverted oxyanion hole structure compared to other SGNH hydrolase family members. Alignment of the Block I and Block III sequences of other SASA structures shows that these features appear universally conserved in the SASA family (Fig. 7B), and presumably adopt the same oxyanion hole structure, as substitution of these residues abrogates activity (López-Cortés et al. 2007). Taken together, this indicates that the unusual oxyanion hole structure observed in *Ec*NanS, *At*4g34215 and CAC0529 delineates the SASA family from other SGNH-hydrolase proteins.

### 1.3 DUF459/PatB family

Although formerly classified as the DUF459 family, structure and function studies have demonstrated that this family comprises the peptidoglycan (PG) *O-*acetyltransferases in Gram-negative bacteria (Moynihan and Clarke 2010). The small DUF459/PatB family currently contains 208 sequences, all of which belong to Gram-negative bacteria. Although not annotated as a member of this family in Pfam, similar SGNH hydrolase domains are also found in Gram-positive PG *O-*acetyltransferases fused to acyltransferase family 3 (AT3) domain, hence classifying them in the SGNH-AT3 family. Interestingly, the *O-*acetylpeptidoglycan esterase (Ape1; Weadge and Clarke 2006) enzyme catalyzing the reverse reaction in these same bacteria is itself classified under GDSL-2, but remains CAZy-unclassified.

PG, the chief component of bacterial cell walls, is a polymer of the β (1 → 4) linked amino sugars *N-*acetylglucosamine (GlcNAc) and *N-*acetylmuramic acid (MurNAc; Fig. 1). These polysaccharides possess a series of short amino acid chains joined to the *O*-lactoyl group at position 3 of MurNAc residues and these are crosslinked together during cell wall assembly, creating a mesh-like network (sacculus) that forms the cell wall. PG is also a potent pathogen-associated molecular pattern (PAMP), which induces the vertebrate innate immune system (Dziarski and Gupta 2005; Wolfert et al. 2007; Martinic et al. 2017) and is the major target of lysozyme, a cornerstone of antibacterial innate immune defense (Mogensen 2009; Yadav et al. 2018). To evade detection and destruction by the host, PG is post-synthetically modified in several ways (Wolfert et al. 2007; Vollmer 2008; Davis and Weiser 2011). Notably, many of these modifications are carried out by other CEs, including the de-*N*-acetylation of GlcNAc or MurNAc by family 4 CEs, which are structurally and mechanistically distinct from the SGNH hydrolase family (Vollmer and Tomasz 2000; Fukushima et al. 2005). O-Acetylation of MurNAc residues, specifically at the C6 hydroxyl position, is a well-established modification that enhances lysozyme resistance in Gram-positive bacteria (Bera et al. 2005) and additionally regulates PG turnover, recycling and pathobiology in Gram-negative bacteria (Fleming et al. 1986; Moynihan and Clarke 2011).

Although there are differences in the pathways for PG O-acetylation in Gram positive and Gram-negative bacteria, both pathways involve the action of an acetyltransferase belonging to SGNH hydrolase family as the terminal step. In both pathways. In Gram-positive bacteria, the peptidoglycan *O*-acetyltransferase A (OatA) enzyme is necessary and sufficient for cell wall acetylation (Bera et al. 2005). In this pathway, an acetyl group from an acetyl coenzyme A donor is transported across the cytoplasmic membrane by the action of the N-terminal integral membrane domain, which belongs to the Acyl_Transf_3 (AT3; PF01757) family. The N-terminal domain of OatA then directly acetylates the C-terminal domain, which belongs to the SGNH-AT3 family and is also discussed here (Jones et al. 2021). In Gram-negative bacteria, the functional equivalents of each OatA domain are present as separate polypeptides. The integral membrane acyltransferase activity is carried out by the peptidoglycan acetyltransferase A (PatA), which belongs to the membrane-bound *O*-acyltransferase (MBOAT; PF03062) family and presumably also accepts an acetyl from a coenzyme A donor. Then, the O-acetylation of PG is carried out by peptidoglycan acetyltransferase B (PatB), the prototypical DUF459 family protein (Moynihan and Clarke 2010). The full mechanism of PG *O-*acetylation mediated by PatA and PatB remains to be demonstrated and it is still unclear if the mechanism discovered of OatA is shared with Gram-negative bacteria. The separation of the membrane-bound and extracytoplasmic acetyltransferases suggests, but does not necessitate, an alternative mechanism.

Although *patB* was originally named *ape2* and was thought to be an acetylesterase, studies of PatB from the pathogen *Neisseria gonorrhoeae* (*Ng*PatB) demonstrated that it in fact functions as an acetyltransferase to modify PG with 6-*O*-acetyl groups at MurNAc residues (Weadge et al. 2005; Moynihan and Clarke 2010). *Ng*PatB was shown to be capable of accepting typical artificial substrates used for the assay of acetylesterases, such as *p*NP-Ac, 4-methylunbelliferyl acetate and α-naphyl acetate, although they displayed a much lower turnover number compared to the true esterases when bulk solvent served as the acceptor (Moynihan and Clarke 2013; Moynihan and Clarke 2014a). Instead, both purified and fragmented cell wall material, or chitooligosaccharides (linear polymers of β-1,4-GlcNAc) could serve as suitable acceptors, and meaningfully increased the turnover number of the enzyme (Moynihan and Clarke 2014a). Though the PatB/DUF459 family enzymes possess the same conserved catalytic machinery as the SGNH hydrolases, a covalent Block I Ser-acetyl intermediate was isolated from *Ng*PatB and demonstrated that the enzyme uses the same conserved mechanism as the hydrolytic enzymes that comprise the majority of the known activities within the SGNH hydrolase family (Moynihan and Clarke 2014b). It remains to be seen what the biological donor of acetyl is to *Ng*PatB. Although acetyl-CoA is presumably an abundant source of acetyl, PatB is localized in the periplasmic space of Gram-negative bacteria where acetyl-CoA would not be available (Moynihan and Clarke 2010). Supporting this notion, PatB is experimentally only weakly active on thioesters (Moynihan and Clarke 2014a). Instead, the MBOAT family PatA is predicted to localize in the inner membrane where it would translocate acetyl from a cytoplasmic acetyl-CoA molecule to the periplasmic space. It has yet to be determined if PatA and PatB would form a direct interaction to transfer acetyl or if this might occur through an acetylated shuttle intermediate (Sychantha et al. 2018a).

### 1.4 SGNH-AT3 family

As the name implies, the SGNH-AT3 family contains sequences that possess an SGNH hydrolase domain appended to an Acyl_Transf_3 (AT3; PF01757) domain. At present, there are 3120 sequences classified as SGNH-AT3, with a majority (2754) belonging to bacteria, with the remainder belonging to animals (478) or oomycetes (75). The only SGNH-AT3 proteins structurally and functionally characterized are the bacterial enzymes OatA from both *Staphylococcus aureus* and *Streptococcus pneumoniae* (Sychantha et al. 2017; Jones et al. 2020) along with OafA and OafB from *Salmonella* spp. (Pearson et al. 2020). Each of these enzymes is known not to function as an acetylesterase, but instead as an acetyltransferase responsible for depositing acetyl groups to surface carbohydrates in bacteria. The OatA enzyme is found in a wide range of pathogenic Gram-positive bacteria and is responsible for O-acetylation of PG in Gram-positive bacteria, much alike the DUF459/PatB family described above. This modification occurs almost exclusively at the C6 hydroxyl of MurNAc residues resulting in resistance of PG to hydrolysis by lysozyme (recently reviewed in (Sychantha et al. 2018a). Not surprisingly, the presence of OatA in the genome of Gram-positive bacteria and the extent of PG O-acetylation correlate with bacterial virulence (Bera et al. 2005). The Oaf proteins, by contrast, are found in Gram-negative bacteria where they have a demonstrated role in the acetylation of lipopolysaccharide, specifically at abequose residues (where the C2 hydroxyl is acetylated by OafA) or rhamnose (where the C2 or C3 hydroxyl groups can be acetylated by OafB) residues of the O-antigen (Fig. 1; (Slauch et al. 1996; Kintz et al. 2015; Pearson et al. 2020). The acetylation of O-antigen by Oaf proteins is serovar-determinant and its presence is required for the production of protective antibodies, at least in the case of *Salmonella typhimurium* infection (Slauch et al. 1995).

**Fig. 8:**
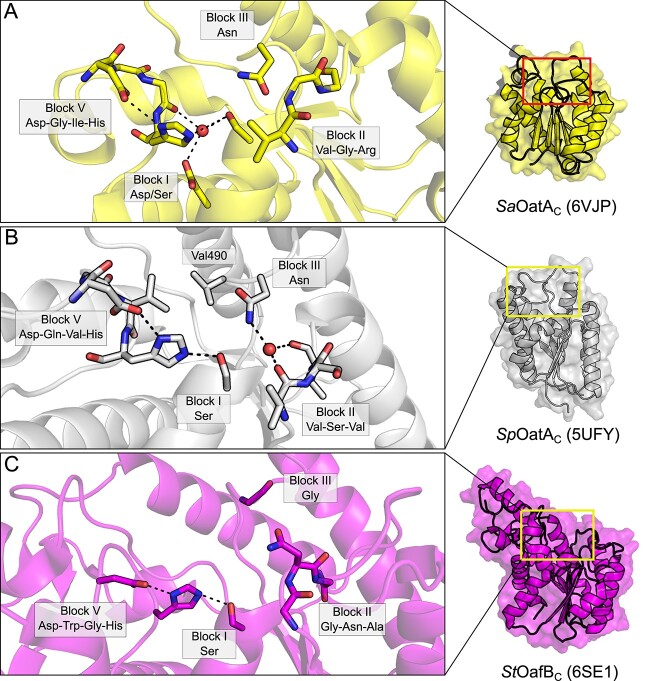
The SGNH-AT3 family. (A) The active site of the *S. aureus* OatA C-terminal domain (*Sa*OatA_C_; 6VJP; yellow) also contains a highly ordered water molecule but coordinated instead by a Block I Asp/Ser pair and a carbonyl group from the Block V loop, which limits solvent access to the active site pocket and promotes transferase activity. (B) The active site of the *S. pneumoniae* OatA C-terminal domain (*Sp*OatA_C_; 5UFY; grey) promotes transferase activity through a highly ordered water molecule at the active site; however, this is achieved through coordination by an inverted Block II loop unique to Streptococci, along with the conserved Block III Asn. Additionally, two Val residues presented by the Block III and V loops form a hydrophobic wall that further limits solvent access to this pocket. (C) The *Salmonella* ser. Paratyphi O-antigen acetyltransferase OafB (*S*tOafB_C_; 6SE1; magenta) possesses an additional helical element and a structured linker domain, which constrain the solvent-accessible surface area of the domain and conferring specificity for acceptor co-substrate(s).

The structures of the C-terminal SGNH domain of OatA from *S. aureus* (*Sa*OatA_C_; 6VJP; Jones et al. 2020) and *S. pneumoniae* (*Sp*OatA_C_; 5UFY; Sychantha et al. 2017) provide important insight into how subtle structural alterations in the otherwise highly conserved SGNH hydrolase domain can not only alter substrate preference, but also shape an entirely new enzymatic activity (Fig. 8). Both proteins adopt a highly similar overall α/β hydrolase fold typical of the SGNH hydrolase family, comprising a central five-stranded β-sheet flanked by seven α-helices (Sychantha et al. 2017; Jones et al. 2020). Interestingly, the two proteins appear to contain unique structural adaptations that promote *O-*acetyltransferase activity (i.e. transfer of the acetyl to carbohydrate rather than bulk solvent) and defined substrate preferences despite their presumptive orthology (Sychantha et al. 2017; Jones et al. 2020). However, it appears that OatA orthologues belong to at least two unique sequence clusters containing either primarily Streptococcal species or else members of genera *Staphylococcus, Listeria, Bacillus, Enterococcus, Lactobacillus* and *Lactococcus*, with each group represented by *Sp*OatA and *Sa*OatA, respectively (Jones et al. 2020). This divergent evolution is then not surprising given the diverse range of Gram-positive genera observed to encode the OatA PG *O*-acetyltransferase.

The structure of *Sa*OatA_C_ demonstrates a typical three-component oxyanion hole comprised of the backbone amide of the catalytic Ser of Block I, the backbone amide of the Block II Gly and the sidechain amide of Asn from Block III (Fig. 8A). The Block III Asn sidechain is much closer to the catalytic Ser than in other SGNH esterases (3.0 Å for *Sa*OatA versus a mean of 5.5 Å and range of 4.8–6.3 Å, for the other esterases discussed here) although this has no obvious mechanistic effect, as replacement of this Asn residue with Ala abrogated all activity like other SGNH hydrolases (Jones et al. 2020). Interestingly, this closer Asn sidechain is also a feature of *Sp*OatA (3.4 Å distance), but not the other transferases discussed here (mean 5.8 Å distance and range of 3.0 to 10.6 Å), probably because of their divergent oxyanion hole arrangements. Additionally, a mechanistic preference for transfer to carbohydrates rather than bulk solvent requires a means to exclude access of solvent to the appropriate face of the acetyl-enzyme intermediate during the catalytic cycle, while maintaining accessibility of the acceptor co-substrate. In *Sa*OatA_C_, this appears to be achieved through the sidechain positioning of a conserved Asp residue, which coordinates a water molecule together with the catalytic Ser residue contained in Block I, as well as the backbone carbonyl of an Ile residue contributed by the Block V loop (Fig. 8A). In support of this proposed mechanism, substitutions of this conserved Asp with either Ala or Asn resulted in a marked increase in both the rate of transfer to solvent (382 and 385 percent of wild-type activity, respectively) or to carbohydrate acceptors (886 and 662 percent of wild-type activity, respectively; Jones et al. 2020). This increase seen in both enzymatic activities with the replacement of the conserved Asp may be due in part to enhanced substrate access and a loss of substrate preference owing to the loss of the coordinated water molecule. Though not a universal feature, even among OatA orthologues, the coordination of a water molecule at this site may be a key structural adaptation of some members in the SGNH hydrolase family that predicts their function as acyltransferases to acceptors other than bulk solvent. Though the number of CEs that have been structurally resolved is limited, structural homology suggests *Sa*OatA_C_ is most alike the resolved structures of *Ct*Ces3–1 and *Tc*AE206, belonging to CE3, and discussed above (Jones et al. 2020).

*Sp*OatA_C_, on the other hand, belongs to a separate clade of OatA orthologues that do not cluster with other sequences outside of genus *Streptococcus* and does not share the particular features of *Sa*OatA_C_ that promote acetyltransferase activity to carbohydrate acceptors (Jones et al. 2020). *Sp*OatA_C_ instead possesses an atypical two-component oxyanion hole, composed of the catalytic Block I Ser backbone amide and the sidechain amide of the Block III Asn (Fig. 8B; Sychantha et al. 2017). The typical conserved Gly of Block II that usually shapes the oxyanion hole is replaced by a Ser residue in *Sp*OatA_C_ (Fig. 8B; Sychantha et al. 2017). Typically, the SGNH hydrolase Block II loop forms a type-II β turn, whereas in *Sp*OatA_C_ the residues of Block II form a type-I β turn. The altered conformation of this loop presents the carbonyl oxygen atoms of two Val residues, immediately N- and C-terminal of the Block II Ser residue, inward toward the active site where they coordinate a water molecule together with the Block III Asn sidechain amide (Fig. 8B). Additionally, two Val residues opposite the active site from the Block II loop create a hydrophobic pocket that lines the face of the active site pocket from where incoming substrate would need to approach, creating a further barrier for bulk solvent to access the active site during intermediate steps of catalysis (Sychantha et al. 2017). *Sp*OatA_C_ and *Sa*OatA_C_ thus rely on similar, but distinct, structural adaptations near the active site to maintain substrate accessibility, while limiting solvent accessibility to the invariant Block I Ser residue—upon which all SGNH hydrolase family members depend upon for catalysis.

The structure of the C-terminal SGNH domain of OafB from *Salmonella* ser. Paratyphi A (*St*OafB_C_; 6SE1; Pearson et al. 2020) demonstrates different structural adaptations toward *O-*acetyltransferase activity. *S*tOafB_C_ contains an extended helical element that is not present in other SGNH-like domains that appears conserved among Oaf proteins (Fig. 8C). The structure also reveals that a linker joining the AT3 and SGNH domains adopts a structured conformation that packs against the SGNH domain. Together, these two structural features appear to constrain access to the active site and result in a reduced solvent accessible surface area compared to other resolved structures of SGNH hydrolases. This finding suggests that specificity for the acceptor co-substrate of lipopolysaccharide is shaped in part by these unique structural elements, corroborated by the observation that truncation of the structured linked domain in *St*OafB_C_ confers activity toward non-native substrates that is not detectable when the linker is present (Pearson et al. 2020).

### 1.5 Hemagglutinin esterase family

Many viruses with tropism for cells of the respiratory or gastrointestinal tract initiate infection and host invasion by binding to sialic acids (Rogers and Paulson 1983). Sialic acids are commonly the terminal residues of cell-surface glycoproteins or other glycoconjugates (Fig. 1) and so differential recognition of these sialic acid subtypes is one form of host cell binding and initiation of viral infectious events (Rogers and Paulson 1983; Angata and Varki 2002; De Groot 2006). However, the sialic acid and/or receptor subtypes for which a virus has specificity may occur frequently in off-target cells (Rogers and Paulson 1983; Matrosovich et al. 2004). The subsequent binding of viruses to these so-called “decoy” receptors, on cells for which these viruses do not possess tropism, is unproductive and necessitates a mechanism for viral release to maintain infectivity. This is achieved through the action of receptor-destroying enzymes (RDEs), which are found throughout toro-, orthmyxo- paramyxo- and coronaviruses, among others (Desforges et al. 2013). These RDEs represent excellent targets for antiviral intervention, as evidenced by the success of Oseltamivir and other related drugs that block the neuraminidase RDE activity of Influenza A and B (Treanor et al. 2000).

The hemagglutinin-esterase (HE) or hemagglutinin-esterase-fusion (HEF) family proteins, named depending upon whether they have functional fusion domains, are common RDEs present exclusively in closely related members of toro-, paramyxo-, orthomyxo- and coronaviruses of mammals and fish (De Groot 2006). These viruses primarily target 9-O*-*acetylated, or less commonly, 4-O-acetylated sialic acids as receptors and so their HEF proteins possess esterase domains with sialate 9-*O*- or 4-*O*-acetylesterase activity (De Groot 2006). The important role played by HE or HEF proteins in infection is corroborated by the observation that catalytically inactive esterase domains of HE proteins abrogate receptor destruction in cell culture and preclude the generation of new infectious viral particles (Zeng et al. 2008; Desforges et al. 2013).

**Fig. 9:**
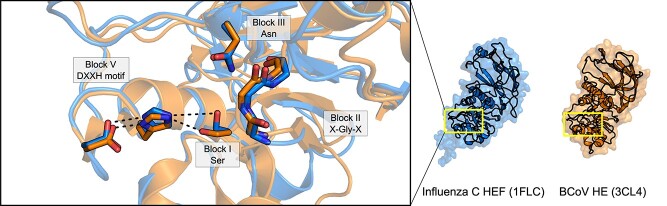
The viral hemagglutinin esterase family. Despite their unusual substrate of *O*-acetyl sialates, the structures of human influenza C (3CL4; orange) and bovine coronavirus (1FLC; aqua) HE and HEF proteins, respectively, contain an SGNH hydrolase family domain with a canonical Ser-His-Asp triad and a family-typical oxyanion hole. The substrate specificity of these HE and HEF proteins is likely conferred by their receptor binding domains.

Pfam currently lists only 22 known family members, with structures of nine unique members available on the PDB. These include the HE/HEF proteins from human influenza C (1FLC; Zhang et al. 1999), H5 avian and H9 swine influenza (1JSM, 1JSD; Ha et al. 2002) as well as bovine coronavirus (BCoV; 3CL4, 3CL5; Zeng et al. 2008). The esterase domain, which itself belongs to the SGNH hydrolase family, is not a continuous and discrete linear portion of the polypeptide chain. The esterase domain is often flanked by the fusion (F) domain, where it is present, and contains the receptor-binding hemagglutinin (R) domain as a linear sequence within it. Despite this unusual architecture, the E domain of HE and HEF proteins still maintain the typical SGNH hydrolase fold, along with a classical Ser-His-Asp catalytic triad spread across Blocks I and V (Fig. 9; Zhang et al. 1999; Ha et al. 2002; Zeng et al. 2008). The oxyanion hole is also composed of the typical backbone amide groups of the Block I Ser and Block II Gly, and the sidechain of the Block III Asn (Zhang et al. 1999; Zeng et al. 2008). This unusual architecture, along with their specificity for host sialosides, defines the hemagglutinin esterases. Additionally, because of their non-continuous amino acid sequences, these E domains align poorly to other SGNH hydrolase family members. Thus, the hemagglutinin esterase family members are not listed as carbohydrate esterases under the CAZy classification system, despite the inclusion of the functionally related SASA family among the CAZy CE database.

Although it would be expected that the esterase domain would contain structural features to accommodate sialic acid, an unusual substrate for CEs, the 9-*O* position is situated distal to the central pyranose ring (Fig. 1). Accordingly, the *O*-acetyl sialoside substrate likely remains exposed during catalysis owing to the small, buried oxyanion hole as the primary point of contact with the enzyme, as seen in most SGNH hydrolase family members discussed here. Instead, the specificity of HE and HEF proteins is attributed to the receptor-binding domain, which folds into a “jelly roll” β-sandwich and generates the required carbohydrate-binding specificity (Rosenthal et al. 1998; Ha et al. 2002; Zeng et al. 2008). Together, these studies show that the utility of the SGNH hydrolase domain is not exclusive of cellular life and shapes yet another role for this diverse family of enzymes.

### 1.6 AlgX family

Biofilms are community structures of cells that encase themselves in a protective matrix. The composition of this matrix is highly variable, but in many bacterial species this matrix is composed chiefly of high-molecular weight exopolysaccharide materials. Alginate is one such exopolysaccharide produced by mucoid *Pseudomonas aeruginosa* (Nivens et al. 2001). Commonly, *P. aeruginosa* can be found in the lungs of cystic fibrosis patients where biofilms composed of alginate pose a serious clinical challenge to treat, and for which patient prognosis is markedly poor. Alginate is principally composed of d-mannuronic (ManA) and l-guluronic acids (Fig. 1). Upon synthesis in the cytoplasm, alginate is translocated to the periplasm where it is subsequently O-acetylated. This O-acetylation, carried out on the C2 and C3 hydroxyl groups of mannuronic residues, is a critical determinant of the biofilm matrix. O-Acetylation has been experimentally shown to enhance biofilm adherence to the lung epithelium, and provides protection from antibiotics and the host immune response (Nivens et al. 2001; Flemming and Wingender 2010; Høiby et al. 2010).

In all, 13 proteins are involved in the biosynthesis, polymerization and export of alginate, 12 of which are located on the *alg*D operon (Chitnis and Ohman 1993). Of these 13 total proteins, 4 are known to function specifically in alginate O-acetylation. These include a protein belonging to the MBOAT family, AlgI; two periplasmic *O*-acetyltransferases, AlgJ and AlgX; and a protein of unknown function, AlgF (Franklin and Ohman 2002;Riley et al. 2013 ; Baker et al. 2014). The topology of these proteins and their predicted functions make them analogous and functionally equivalent to the process of peptidoglycan O-acetylation, whereby AlgI and AlgJ/AlgX are analogous to the two-protein systems of PatA/PatB in Gram-negative bacteria, and the OatA_N_/OatA_C_ domains found in Gram-positive bacteria (Sychantha et al. 2018a).

The ability for AlgJ and AlgX to function as *O-*acetylesterases permitted their biochemical characterization in vitro using the surrogate acetyl donor 3-carboxyumbelliferyl acetate (Riley et al. 2013; Baker et al. 2014). AlgJ from *P. aeruginosa* and *Pseudomonas putida* (*Pa*AlgJ and *Pp*AlgJ) and AlgX from *P. aeruginosa* (*Pa*AlgX) all displayed weak esterase activity with similar *K*_m_, and interestingly *Pa*AlgX displaying 3-fold greater catalytic efficiency (*k*_cat_/*K*_m_). Moreover, *Pa*AlgJ and *Pa*AlgX were compared for their ability to interact with ManA oligomers between 4–12 residues in length (ManA_4_ – ManA_12_). *Pa*AlgJ showed extremely weak binding to ManA_4_ – ManA_7_ with *K*_a_ values less than 500 M^−1^, and no binding of oligomers greater than 7 units in length. On the other hand, *Pa*AlgX bound to all oligomer chains, with increasing *K*_a_ values proportionate to increasing chain length; values ranging from 1.0 ± 0.5 × 10^3^ M^−1^ to 19.0 ± 0.3 × 10^3^ M^−1^ for ManA_4_ to ManA_12_, respectively. No specific binding was observed for *Pa*AlgX with undeca- and pentadeca-hyaluronic acid confirming substrate specificity for alginate oligomers rather than any acidic oligosaccharide. Further, *Pa*AlgX is able to O-acetylate alginate in vitro using *p*NP-Ac and 3-carboxyumbelliferyl acetate as surrogate acetyl donors, and a polymannuronic acid decamer (ManA_10_) as an acetyl acceptor. Neutral, commercially available sugars, cellohexose, xylohexose and maltotriose were not O-acetylated in the presence of *Pa*AlgX, supporting the substrate specificity to alginate. The differences in binding and acetylesterase ability between AlgJ and AlgX suggests that AlgX is the only enzyme involved with the direct O-acetylation of alginate in vivo, in a non-redundant successive mechanism with the other proteins involved.

**Fig. 10:**
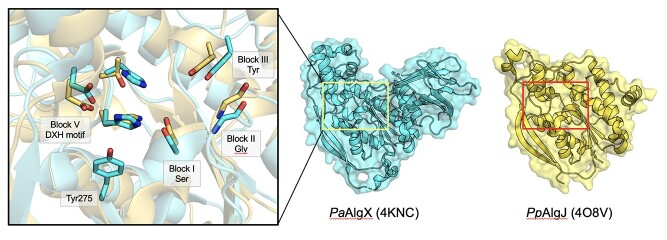
The AlgX family enzymes. Enzymes from this family adopt a circularly permuted SGNH fold in the order of HSGN. The active sites of *P. aeruginosa* AlgX (*Pa*AlgX; 4KNC; cyan) and *P. putida* AlgJ (*Pp*AlgJ; 4O8V; yellow) differ from the typical SGNH hydrolases utilizing the side chain hydroxyl group of a Block III Tyr residue, and a Block V DXH motif. An additional Tyr residue on *Pa*AlgX (Tyr275) presumably aids in substrate binding and positioning in the active site. RMSD: 2.1 Å over 165 C-alpha atoms.

*Pp*AlgJ (4O8V; Baker et al. 2014) and *Pa*AlgX (4KNC; Riley et al. 2013) have both been structurally characterized, each exhibiting SGNH hydrolase-like structures (Fig. 10). The two proteins share 69 percent and 30 percent sequence similarity and identity, respectively (Franklin and Ohman 1996). The hydrolase domains align well using DALI with an RMSD of 2.1 Å over 165 Cα atoms (Baker et al. 2014). Similar to other SGNH hydrolase proteins, AlgJ and AlgX utilize a Ser-His-Asp catalytic triad and oxyanion hole dependent mechanism, the residues of which are spread across the consensus Block motifs I-III and V. While the spatial arrangement of the active site residues are conserved, both AlgJ and AlgX are circularly permutated, leading to a sequential rearrangement of the Block motifs in the order of H-S-G-N compared to the canonical S-G-N-H arrangement typical of the SGNH superfamily (Baker et al. 2014). While *Pp*AlgJ and *Pa*AlgX are considered SGNH hydrolase-like proteins, and aside from the aforementioned circular permutation of the active site residues, they both have key differences to the typical SGNH hydrolase fold, including (i) replacement of the Block III Asn with a Tyr residue; (ii) Block I GTSYS consensus motif compared to the typical GDSL(S) motif; and (iii) Block V DXH motif instead of the typical DXXH motif where the catalytic acidic residue is typically located three residues upstream of the His (Fig. 10; Riley et al. 2013; Baker et al. 2014). Catalytic variants of *Pa*AlgJ: D193A, H195A and S297A, and *Pp*AlgJ: D190A, H192A and S288A, each reduced in vitro esterase activity by 80 percent, whereas similar catalytic similar variants in *Pa*AlgX: D174A, H176A and S269A completely abrogated esterase activity in vitro and alginate O-acetylation in vivo. Additionally, an Ala variant of the Block III Tyr in *Pa*AlgX (Y328A), as well as a Y275A variant, reduced alginate O-acetylation by 50 percent and 40 percent, respectively (Riley et al. 2013). These residues are positioned on either side of the catalytic triad (Fig. 10), and presumably aid in substrate binding and positioning in the active site, rather than contribution to the oxyanion hole (as aromatic residues frequently bind to sugar molecules via hydrophobic interactions (Boraston et al. 2004). Additional differences of *Pp*AlgJ and *Pa*AlgX to the typical SGNH hydrolase fold include (iv) a 4-stranded core β-sheet instead of a 5-stranded core β-sheet typical of the SGNH hydrolases, where the fifth strand is replaced with an isolated β-bridge (F168 in *Pp*AlgJ; W153 in *Pa*AlgX); (v) two long, antiparallel β-strands present across one side of the protein (β4, β5); and (vi) a “cap” domain atop the active site comprised of two short antiparallel β-strands (β1, β2), a series of short α-helices (α2 in *Pp*AlgJ; α1, 3, 4, 7 in *Pa*AlgX), and five 3_10_ helices in *Pp*AlgJ (*t*1–3, *t*5–6) (Riley et al. 2013; Baker et al. 2014). While the exact function of the two long antiparallel β-strands is currently unknown, it is speculated that they are involved with protein–protein interactions, given the involvement of other proteins in the system.

*Pp*AlgJ and *Pa*AlgX have significantly different pairwise structural features. Unique to *Pp*AlgJ is a shallow electronegative groove that runs across the surface and around the active site, as well as two AlgJ signature motifs (Baker et al. 2014). The shallow groove contains the Block V catalytic residues D190 and H192, which is also located in the cap domain, as well as other residues which are well conserved in six other *Pseudomonas* sp. and *Azobacter vinelandii*. The two conserved sequence motifs of *Pp*AlgJ, termed “AlgJ signature motifs”, have been recognized in other homologs of *Pp*AlgJ (Franklin et al. 2004), and include the conserved motifs of ΦΦΦPXK (Φ represents any hydrophobic residue; residues 129–134), and (R/K)TDTHW (residues 188–193) that contains the catalytic Block V Asp and His residues (Baker et al. 2014). Replacement of residues within each of these signature motifs leads to the impairment or ablation of alginate O-acetylation in vivo (Franklin et al. 2004). Notably, there are two intramolecular interaction networks, of which residues from the signature motifs are involved in (Baker et al. 2014). The first network in *Pp*AlgJ comprises the residues K134, T189, D190 and H192, which form a H-bonding network with residues L187, D254 and the nucleophilic S288. The second intramolecular network is composed of a series of hydrophobic interactions centered around the completely buried and conserved core hydrophobic residue W193, also involving V131, Y289, W295 and F297. *Pa*AlgJ variants of residues P135A, K137A, D193A, H195A and W196F (P132, K134, D190, H192 and W193 in *Pp*AlgJ) ablate O-acetylation in vivo (Franklin et al. 2004). In *Pp*AlgJ, it is expected that the P132A variant would alter the structure and proper function of K134, where a K134A variant disrupts the H-bonding network associated with D190, which would disrupt the positioning of the DXH motif and impair catalysis. Additionally, the W193A variant of *Pp*AlgJ is expected to disrupt the hydrophobic interaction network and cause structural perturbations in close proximity to the catalytic H192. Variants impacting the structural integrity and intramolecular interactions involved with these key residues would disrupt proper catalysis by increasing the p*K*a of the nucleophilic Block I Ser and altering its orientation within the active site (Baker et al. 2014).

*Pa*AlgX contains a deep electropositive groove which has been shown to be compatible for binding of the anionic alginate polymer (Riley et al. 2013). Moreover, *Pa*AlgX contains a second region of electropositive charge which is located on a C-terminal Type-B CBM domain, and harbors a β-sandwich jelly roll fold. Both distinct regions of electropositive charge contain residues critical for the binding and/or catalysis of alginate O-acetylation. Notably, the presence of a CBM on *Pa*AlgX may explain why it is able to bind alginate oligomers, unlike *Pp*AlgJ that does not possess a CBM (Baker et al. 2014). The electropositive region on the C-terminal CBM domain is formed by several residues and creates a distinctive “pinch point” where the alginate polymer passes (Riley et al. 2013). A series of aromatic residues interact with rings on the polymeric sugar, leading to stabilization of the polymer-protein complex and defined substrate specificity. The pinch point may also conceivably bind alginate along a path that joins the polymer between the conserved residues of the pinch point, to a positively charged region in close proximity to the active site residues. Polar residues located on a groove on the surface of the CBM domain further increase the stability of the polymer-protein complex through hydrogen-bonding interactions. Included in the pinch point is a surface-exposed W400 residue that is completely conserved across all aligned *Pseudomonas* spp. and *Azotobacter vinelandii*. A second surface-exposed residue, T398, is highly conserved among alginate-producing species. Additionally, located beside W400, on the opposite side of the groove, there is two residues, R364 and R406, which are presumed to aid in the binding of the alginate polymer. R406 is conserved in charge among alginate-producing species, replaced only by a histidine. In all aligned species other than *A. vinelandii*, a basic or polar residue is found in the equivalent position of R364. Lastly, two Lys residues, K396 and K410, are located just outside of the pinch point, and are proposed to direct alginate along the face of the CBM. K396 is conserved in charge in all aligned species except for *A. vinelandii* and *P. mendocina*, and K410 is completely conserved except in *A. vinelandii*. Distinctively, *Pa*AlgX also contains two disulfide bonds. The first is between C44 and C229 which positions the two long β-strands (β4, β5) alongside the N-terminal hydrolase domain. The second is between C347 and C460 in the C-terminal CBM domain, which appears to fix the relative orientation of the two domains with respect to one another, further supporting its role in alginate binding/catalysis.

### 1.7 DHHW family

Of the eleven closely related species of the Gram-positive *Bacillus cereus* group of pathogens, *B. cereus* and *Bacillus anthracis* are the most common for causing disease in humans (Drobniewski 1993; Liu et al. 2015). The surface layer (S-layer) of these two pathogens consists of several S-layer-associated proteins (BSLs), including surface array protein (Sap) and extractable antigen 1 (EA1) that remodel peptidoglycan and contribute to growth and survival, maintenance of the cell, as well as interactions with the host immune system leading to pathogenesis (Mesnage et al. 1997; Fagan and Fairweather 2014). Secondary cell wall polysaccharides (SCWPs) are anchored to the PG sacculus and extend outward, forming a scaffold upon which the S-layer self-assembles (Leoff et al. 2008; Fouet 2009).SCWPs are comprised of repeating units of *N*-acetylmannosamine (ManNAc), *N*-acetylglucosamine (GlcNAc) and *N*-acetylgalactosamine (GalNAc; viz. (→4) β-ManNAc (1 → 4) β-GlcNAc (1 → 6) α-HexNAc (1→) trisaccharide (where HexNAc represents either GalNAc or GlcNAc; Fig. 1), and terminated by a 4,6-pyruvyl-β-D-galactosaminyl residue. The O-acetylation of SCWP has been recognized as an essential step for the self-assembly of S-layer proteins to the cell wall (Lunderberg et al. 2013). PatB1 from *B. cereus* (*Bc*PatB1) has been characterized as a SCWP *O*-acetyltransferase and represents the first enzyme of the DHHW family of the SGNH hydrolase superfamily of proteins (Sychantha et al. 2018b). Whereas MBOAT proteins are not known to catalyze the acylation of extracellular wall components like SCWP, the putative MBOAT protein PatA1 from *B. cereus* (*Bc*PatA1) was identified to play a role in SCWP O-acetylation together with *Bc*PatB1 (Lunderberg et al. 2013; Sychantha et al. 2018b), analogous to the PatA/PatB system in *N. gonorrhoeae* for the O-acetylation of PG (Sychantha et al. 2018a).

*Bc*PatB1 was biochemically characterized in vitro for its ability to catalyze the hydrolysis of *p*NP-Ac (Sychantha et al. 2018b). Notably, the specific activity of *Bc*PatB1 hydrolyzing *p*NP-Ac was three orders of magnitude lower than that of true PG esterases, such as Ape1 from *N. gonorrhoeae* (*Ng*Ape1; Weadge and Clarke 2006). *Bc*PatB1 was also tested for its ability to utilize acceptor ligands, with *p*NP-Ac as the acetyl-donor, to confirm it as a true acetyltransferase (Sychantha et al. 2018b). It was not able to O-acetylate chitosan pentamer (GlcN_5_) or GlcNAc; however, it was observed to acetylate disaccharides of GlcNAc-(β-1,4)-MurNAc and GlcNAc-(β-1,4)-MurNAc-l-alanine-d-isoglutamine (GMDP). Moreover, acetyltransferase activity was found to be 3-fold higher than esterase activity, confirming *Bc*PatB1’s role as an acetyltransferase. Distinguishing its role from *Ng*PatB, *Bc*PatB1 did not produce any *O*-acetyl-PG, and the only acetylated residue of acceptor ligands was GlcNAc, as opposed to MurNAc as seen for *Ng*PatB. Differing degrees of polymerization (DP) were tested for acceptor chitooligomers; however, there were no trends observed with increasing DP, suggesting that *Bc*PatB1 is an exo-acting *O*-acetyltransferase, binding only two residues in the active site. Furthermore, the location of the *patB1* gene is between *patA1* and the *csaB-sap-eag* S-layer genes in *B. cereus*, suggesting *Bc*PatB1 is a true SCWP *O*-acetyltransferase (Sychantha et al. 2018b). To confirm whether SCWP is the natural substrate for *Bc*PatB1, a trisaccharide comprised of β-ManNAc-(1–4)-β-GlcNAc-(1,6)-α-GlcNAc (MGG) was synthesized and tested as an acetyl-acceptor ligand, using *p*NP-Ac as the acetyl-donor. Indeed, *Bc*PatB1 exhibited 1.5 times greater catalytic efficiency (*k*_cat_/*K*_m_) than for GlcNAc_3_, and only the C3 hydroxyl of the β-GlcNAc residue of MGG was O-acetylated, consistent with the known site of O-acetylation of natural SCWP from various species of pathogenic *Bacilli* (Leoff et al. 2008).

**Fig. 11:**
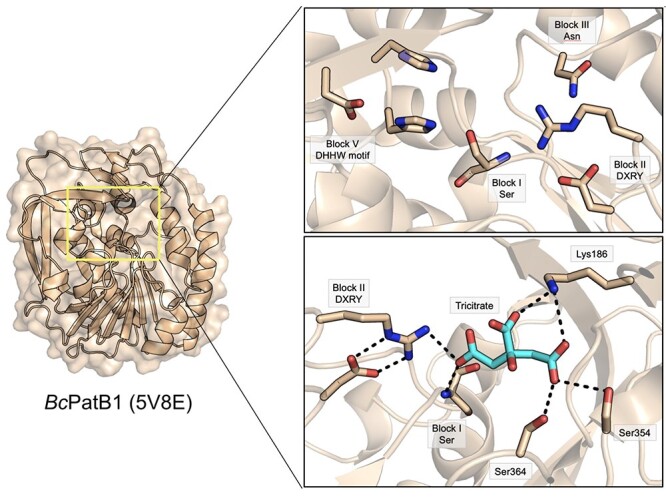
The DHHW family. (A) *B. cereus* PatB1 (*Bc*PatB1; 5V8E; brown), the only structurally resolved member, is structurally similar to the AlgX family with a circularly permuted HSGN fold, possessing an α/β/α topology comprised of a core 8-stranded β-sheet with a 100° twist, surrounded by 11 α-helices. (B) The active site of *Bc*PatB1 comprises sequence block motifs that differ from the canonical SGNH hydrolases including a Block II DXRY motif, and a Block V DHHW motif. (C) The side chain carboxylate group of the Block II Asp residue positions the side chain guanidino group for its role in the oxyanion hole. A tricitrate molecule (cyan) was co-crystallized with *Bc*PatB1, forming hydrogen bonds with Lys186, Ser354 and Ser364. These residues are located at the closed end of the active site groove and presumably aid in substrate binding and positioning.

The crystal structure of *Bc*PatB1 (5V8E; Sychantha et al. 2018b) reveals an α/β/α topology, comprised of a core 8-stranded β-sheet with a 100° twist, surrounded by 11 α-helices (Fig. 11A). The closest functionally characterized structural homologs of *Bc*PatB1 are *Pp*AlgJ and *Pa*AlgX from the AlgX family of proteins (Riley et al. 2013; Baker et al. 2014), aligning with an RMSD of 2.4 Å over 195 equivalent Cα atoms, and 3.17 Å over 197 equivalent Cα atoms, respectively. Similar structural elements between the AlgX family and *Bc*PatB1 include (i) two antiparallel β-strands; (ii) a groove along the surface of the protein; and (iii) a circularly permutated SGNH core consisting of the Ser-His-Asp catalytic triad spread across Blocks I and V typical of SGNH hydrolases. Interestingly, *Bc*PatB1 comprises sequence block motifs that differ from the canonical SGNH hydrolases, including: Block I KDS, Block II DXRY and Block V TDHHW (Fig. 11B), where bolded residues represent invariant residues among PatB1 homologs identified in 56 unique bacteria (all from *Bacilli* and *Clostridia*, including the SCWP-producing *Clostridium difficile*) (Sychantha et al. 2018b). The S from the KDS motif together with the D and H from the TDHHW motif represent the catalytic triad residues.

The two antiparallel strands in *Bc*PatB1 (β5 and β8) are positioned along the surface on the back side of a groove opening and are stabilized by a disulfide bridge between Cys240 and Cys266. The groove of *Bc*PatB1 consists of an electronegative open end, and an electropositive closed end, the latter of which interacts with the negatively charged carboxylate of the terminal ketyl pyruvyl group of natural SCWP. The closed end of this groove is a particularly important structural feature of *Bc*PatB1 as the enzyme acts exclusively on the terminal end of natural SCWPs in an *exo-*acting mechanism. A tricitrate molecule was co-crystallized in the closed end of the groove, making hydrogen bonds with Lys186, Ser354, Ser 364 and the nucleophilic Ser337 (Fig. 11C). Importantly, Ser337 is located 20 Å away from the closed end of the groove, providing sufficient room to accommodate the terminal 4,6-pyruvyl-β-D-galactosaminyl residue of SCWP and to specifically position the C3 hydroxyl of the β-GlcNAc residue for O-acetylation. *Bc*PatB1 differs from the AlgX proteins mainly by a unique 80-residue addition (residues 276–322) that is formed by β9-β11, and forms 3 antiparallel β-strands that extend the core β-sheet past β12, lengthening the active site groove by 14 Å relative to the canonical active site of SGNH hydrolases. Moreover, as found in the AlgX family, *Bc*PatB1 does not contain a CBM (as found in AlgX), or an α-helical cap domain (Riley et al. 2013; Sychantha et al. 2018b). Rather, *Bc*PatB1 contains a unique β-sandwich-like cap domain formed by β1 and β2 (Sychantha et al. 2018b).

Located at the closed end of the groove, *Bc*PatB1 contains a circularly permutated active site that maintains the canonical spatial arrangement of the catalytic triad residues as found in typical SGNH hydrolases. Ser337, His202 and Asp200 make up the catalytic triad of *Bc*PatB1. H202A and D200A variants reduced O-acetylation of GlcNAc_2_ in vitro, where an S337A variant completely abolished activity. To support S337 as the catalytic nucleophile, a sulfonyl adduct of Ser337 was crystallized (5V8D; Sychantha et al. 2018b), and all activity was completely abrogated when *Bc*PatB1 was incubated with methanesulfonyl fluoride, a known mechanism-based inhibitor of serine proteases, esterases and lipases. Additionally, a H201A variant reduced esterase activity to 52 percent, but completely abolished transferase activity in vitro, suggesting its role to likely aid in productive binding of the substrate in vivo. Unique to *Bc*PatB1, the oxyanion hole does not utilize the role of the Block III Asn residue, but rather uses the sidechain guanidinium group of the Block II Arg359 and the backbone amide of the nucleophilic Ser337 (Fig. 11C). The Block III Asn386 is not conserved in PatB1 homologs, and is displaced 6 Å away from Ser337, deterring any chance of its participation in oxyanion hole stabilization. Instead, the invariant Block II Arg359 in PatB1 homologs, replacing the Block II Gly of typical SGNH hydrolases, is found on a loop between β8 and α11. The Arg359 residue is anchored by a salt bridge between its guanidinium group, and the side chain O’s of Asp357, and makes H-bond contacts with two divalent O’s of the sulfonyl adduct. Furthermore, an R359A variant abolished esterase and transferase activity, whereas an R359K variant had some residual activity, supporting the proposed role of Arg359 in the oxyanion hole. Additional H-bonds are located between the O2 of the sulfonyl adduct with the backbone amide of the nucleophilic Ser337.

The unique features displayed by *Bc*PatB1 make it the first characterized enzyme of the DHHW subfamily of SGNH superfamily of hydrolases (Sychantha et al. 2018b). A second member of the DHHW family, EUBSIR_00411 produced by *Eubacterium siraeum,* has been structurally resolved (4NZK), but this protein was not biochemically characterized. EUBSIR_00411 contains 3 antiparallel β-strands compared to only 2 antiparallel β-strands in *Bc*PatB1 (β1 and β2 in *Bc*PatB1), each located at the N-terminus (Sychantha et al. 2018b). EUBSIR_00411 contains an α-helical cap domain comprised of two α-helices, similar to the AlgX proteins, whereas β1 and β2 of *Bc*PatB1 form a β-sandwich-like cap domain. This domain is likely to aid in the specific productive binding of an exopolysaccharide substrate.

### 1.8 The PC-Esterase Family

**Fig. 12:**
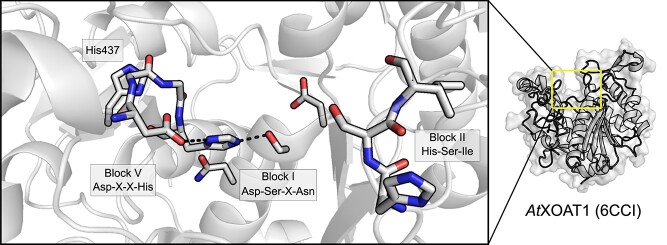
The PC-Esterase family. The structure of *At*XOAT1 (6CCI), a xylan 2-*O*-acetyltransferase found in the Golgi of plants, demonstrates an SGNH hydrolase fold with a deep active site cleft and a unique oxyanion hole.

The PC-Esterase family is comprised of 10,956 members belonging exclusively to Eukarya, namely plants, fungi and some vertebrates. Known members include Cas1p from *Cryptococcus neoformans*, which is required for capsular polysaccharide O-acetylation, a critical virulence factor for survival of *C. neoformans* in the host (Janbon et al. 2001). Many plant proteins known to function in freezing, cold, drought, or salt resistance are included in this family, typified by *ESKIMO*1 from *A. thaliana* (Anantharaman and Aravind 2010; Bischoff et al. 2010; Lefebvre et al. 2011). Although they are generally understood to function as Golgi-localized polysaccharide *O*-acetyltransferases that act on cell-surface glycans or glycoproteins, there is a dearth of available structure/function studies on enzymes belonging to this family. Only XOAT1 from *A. thaliana (At*XOAT1) has an available structure and detailed biochemical characterization (Lunin et al. 2020). This enzyme is responsible for 2-O-acetylation of xylan (Fig. 1), carried out by the C-terminal PC-Esterase domain, with an N-terminal variable region and predicted transmembrane helix that would plausibly anchor the protein in the Golgi membrane. The crystal structure of this enzyme (6CCI; Fig. 12) reveals a typical Ser-His-Asp catalytic triad presented to the active center by Blocks I and V. The active site is shaped by a deep groove that is less common of SGNH hydrolases in general. While the Block II region folds into the usual loop structure, it appears displaced in XOAT1 and does not contribute to the oxyanion hole. Instead, an Asp and Asn residue (Asp^215^/Asn^220^) near the Block I Ser and an additional His residue (His^437^) near the Block V DXXH motif each appear to contribute to the active site as suggested by genetically-engineered site-specific replacements. Surprisingly, the Block III helix that would normally present the critical Asn instead contains a stretch of hydrophobic residues, perhaps in an analogous way to the hydrophobic Val residues seen in OatA that may occlude solvent access and promote the transfer of acetyl groups to carbohydrate substrates rather than bulk solvent.

### 1.9 Non-carbohydrate esterases

Although many members of the SGNH hydrolase family studied to date are carbohydrate-active, structural and functional studies have shed light on family members with other enzymatic activities and exemplify the remarkable flexibility of this catalytic fold. The mammalian platelet-activating factor (PAF) is a phospholipid messenger with various known roles in neuronal migration and inflammatory or thrombotic responses (Kelesidis et al. 2015). PAF is constitutively produced and secreted by various cell types and its activity is controlled by the action of platelet activating factor acetylhydrolases (PAF-AHs), known SGNH hydrolases (Ho et al. 1997). Indeed, the structure of PAF-AH complexes (1WAB; Ho et al. 1997; 1FXW; Sheffield et al. 2001) reveal a typical SGNH hydrolase fold bearing the usual Ser-His-Asp catalytic triad shared between Blocks I and V, along with an oxyanion hole composed of the Block I Ser, Block II Gly and Block III Asn. These enzymes function in deacetylation, and thus, inactivation of the phospholipid messengers and play an important role in control of their activity.

Perhaps the most remarkable SGNH hydrolase structure discussed here is the esterase from *Streptomyces scabies* (*Ss*Est; 1ESC; Wei et al. 1995), the causative bacterial agent of potato scab disease. The esterase is thought to function in the hydrolytic breakdown of suberin, a water-impermeable polyester material found in various plant tissues, including potato tubers, and this enzymatic breakdown would facilitate the invasion of plant tissues by *S. scabies* (Komeil and Beaulieu 2013). The crystal structure of *Ss*Est displays a typical SGNH fold bearing many large, extended random coil structures on the C-terminal face of the central β-sheet. These loops fold back against the active site pocket and shape a large and chiefly hydrophobic face of the enzyme, where incoming substrate would approach and be accessible only through a deep channel (Wei et al. 1995). The structure of *Ss*Est features all three cysteine pairs in the sequence forming disulfide bonds, a feature presumably linked to the enzyme’s thermotolerance, generally an unusual feature of other SGNH hydrolases (Wei et al. 1995). Although a typical oxyanion hole composed of H-bond donors from Blocks I-III is present, the catalytic Block V acid is not present, but instead the catalytic Block V His forms a hydrogen bond with the carbonyl group of a Trp residue that occupies the equivalent location to the conserved Block V Asp or Glu (Wei et al. 1995), a feature apparently shared with CE2 that may have distant homology to *Ss*Est.

A testament to true flexibility, the multifunctional thioesterase I/protease I/lysophospholipase L_1_ from *Escherichia coli,* formerly named TesA/ApeA/PldC (now TAP) is an SGNH hydrolase family member with demonstrated thio- and arylesterase (Lee et al. 1997), protease (Pacaud and Uriel 1971) and phospholipase activities (Albright et al. 1973). The resolved crystal structure (1IVN; Lo et al. 2003) also demonstrates an unremarkable SGNH hydrolase bearing all of the typical conserved Block I-V features. Although its biological role is a subject of contention and was initially described separately by three different names, its cellular location in the periplasm and relative activities on these putative substrates suggest that the natural substrate of TAP is most likely a phospholipid (Lo et al. 2003). The discovery that TAP is a highly substrate-promiscuous bacterial lipase demonstrates the likely divergent evolution of early SGNH hydrolase ancestors directly from the α/β hydrolase family.

### 1.10 Summary

Since its first delineation and structural resolution in 1995, the SGNH hydrolase family has unfolded in the literature as a template for the metabolism of a wide variety of ester-substituted substrates, namely carbohydrates. The ubiquitous SGNH hydrolase domain has utility in the maintenance and turnover of the plant cell wall xylans, rhamnogalacturonans and mannans; of receptor destruction of sialoside glycoproteins; of the bacterial cell wall peptidoglycan and secondary cell-wall polysaccharides in some bacteria; the biofilm exopolysaccharide alginate; of the lipopolysaccharide O-antigen in Gram-negative bacteria; and of non-carbohydrate substrates suberin, platelet-activating factor and phospholipids. These important biocatalysts are found ubiquitously across all domains of life, and have important demonstrated roles in mammals, plants, fungi and bacteria. These enzymes are known to catalyze key steps in cellular signaling events, or in the establishment of symbiosis or pathogenicity, making them biochemically rich and important targets for industrial biofuel development, carbohydrate bioconversion, and for new strategies to combat disease in humans, livestock, or economically important crops. These individual studies highlight how this wide variety of biological activities are possible through subtle alterations of the single SGNH hydrolase catalytic fold and may help predict many new functions for this family that are yet to be discovered.
